# Structural insights into the activation of phospholipase D1 by the small GTPase ARL11

**DOI:** 10.1016/j.jbc.2026.113375

**Published:** 2026-07-28

**Authors:** Douglas J. Marr, Shujuan Gao, Michael A. Frohman, Michael V. Airola

**Affiliations:** 1 Department of Biochemistry and Cell Biology, Stony Brook University, Stony Brook, New York, USA; 2 Department of Pharmacological Sciences, Stony Brook University, Stony Brook, New York, USA

**Keywords:** phospholipase D, small GTPase, ARF-like GTPase 11, phosphatidylcholine, phosphatidic acid, liposome, high-performance liquid chromatography (HPLC), enzyme purification, enzyme mutation

## Abstract

Phospholipase D1 (PLD1) is a lipid-metabolizing enzyme that produces phosphatidic acid (PA) and contributes to pathologies such as cancer, neurodegeneration, and cardiovascular diseases. PLD1 is known to be activated by Rho GTPases at a well-defined site, and by ARF GTPases at a separate, poorly defined site. Recently, the ARF-like GTPase 11 (ARL11) was identified as an activator of PLD1, but the mechanism by which ARL11 stimulates PLD1 activity remained unknown. Here, we use *in vitro* biochemical assays and structural predictions to provide insight into PLD1 activation by ARL11. We find that ARL11 must be loaded with GTP to stimulate PLD1 and corroborate that the loop within the catalytic domain of PLD1 is necessary for this stimulation. Leveraging AlphaFold 3, we predicted the structure of an ARL11-PLD1 complex. Point mutants of ARL11 at the predicted PLD1-ARL11 interface disrupted PLD1 stimulation, confirming the orientation of ARL11 in complex with PLD1. The N- and C-terminal ends of the PLD1-specific loop were predicted to be disordered in the absence of ARL11 but were predicted to adopt secondary structure elements that interact with ARL11 in the complex. Internal deletion mutants of PLD1 that retained these elements, but removed the remainder of the loop, were sufficient for ARL11 stimulation. These findings suggest that ARL11 interacts with PLD1 using three adjacent, biochemically distinct interfaces, and that the ends of PLD1’s disordered loop form ordered structures to facilitate these interactions. Overall, this advances our structural understanding of the activation of PLD1 by the small GTPase ARL11.

Phospholipase D1 (PLD1) is a peripheral membrane enzyme (1) that localizes to intracellular cell membranes and translocates, upon activation, to the inner leaflet of the plasma membrane (2, 3), where it catalyzes the hydrolysis of phosphatidylcholine (PC) (4) to produce phosphatidic acid (PA) and choline as products (2). PA in particular is a bioactive signaling molecule (5) and is upstream of signaling proteins such as phosphatidylinositol 4-phosphate 5-kinase (PI4P5K) (6, 7), mammalian target of rapamycin (mTOR) (6, 8), rat sarcoma virus GTPase (RAS) (9), and extracellular signal-regulated kinases 1 and 2 (ERK1/2) (9). Through the generation of PA, PLD1’s activity can impact a vast array of cellular processes, including endocytosis (5), exocytosis (5), cytoskeletal reorganization (10), cell migration (11), cell proliferation (12, 13), anti-apoptotic signaling (14), cell cycle progression (5), and transcriptional regulation (5).

PLD1’s activity has been implicated in numerous disease pathologies, such as Alzheimer’s disease (15, 16), autoimmune disease (17, 18), cardiovascular disease (19), viral infection (20), and cancer. PLD1’s role in cancer has been especially well-studied, where its activity has been found to contribute to tumorigenesis (9, 12, 21), tumor angiogenesis (12), metastasis (9, 12, 21), tumor growth (21), tissue invasion (21), and apoptotic signal resistance (22), prompting research into PLD1’s regulation (4, 23, 24, 25, 26, 27, 28) and inhibition (5, 14, 20, 29, 30, 31, 32, 33).

Unlike human Phospholipase D2 (PLD2), which catalyzes the same reaction, PLD1’s basal activity is low (34, 35), and it is sensitive to stimulation by upstream effectors (4, 28, 35, 36). Canonically, these effectors are the small GTPases ADP-riboslyation factor 1, 3, 4, 5, and 6 (ARF1, ARF3, ARF4, ARF5, and ARF6) (4, 25, 26, 36, 37, 38), cell division control protein 42 homolog (CDC42) (28, 39), Ras homolog family member A (RhoA) (28), and Ras-related C3 botulinum toxin substrate 1 (Rac1) (40); and protein kinase C (PKC) (35). However, it was recently discovered that Arf-like GTPase 11 (ARL11) and Arf-like GTPase 14 (ARL14), two less studied members of the ARF family of small GTPases (41), colocalize with PLD1 when loaded with GTP, and stimulate PLD1’s activity *in vitro* (42). This stimulation depends on a loop of approximately 150 amino acids present between the two tandem HKD domains in PLD1’s catalytic domain. The loop was previously predicted to be entirely disordered and is absent from PLD2 (2, 43), but, aside from its dependence on PLD1’s loop, the mechanism by which ARL11 and ARL14 stimulate PLD1 remained unknown.

Here, we explore the mechanism of ARL11’s activation of PLD1. We corroborate prior findings that PLD1’s disordered loop is necessary for its stimulation by ARL11, and demonstrate that ARL11 requires GTP to activate PLD1. Using AlphaFold 3 (44) predictions with experimental validation, we define the surfaces and regions within PLD1 and ARL11 involved in ARL11’s activation of PLD1. Altogether, this advances our understanding of PLD1 regulation by small GTPase activators.

## Results

### Establishment of a novel PLD1 activity assay

To study the interaction of PLD1 and ARL11, we developed a PLD activity assay that separates fluorescently labeled PC (NBD-PC) from fluorescently labeled PA (NBD-PA) while simultaneously measuring the fluorescence of each using high-performance liquid chromatography (HPLC) coupled with a fluorimeter. Additionally, the NBD-PC substrate used in this assay is delivered in liposomes to better mimic PLD1’s native membrane environment relative to micelles. These liposomes are composed of a 45:45:5:5 molar ratio of 1-palmitoyl-2-oleoyl-glycero-3-phosphocholine (POPC), 1-palmitoyl-2-oleoyl-sn-glycero-3-phosphoethanolamine (POPE), 1-Myristoyl-2-[12-[(7-nitro-2-1,3-benzoxadiazol-4-yl)amino]dodecanoyl]-sn-Glycero-3-Phosphocholine (NBD-PC) and brain L-α-phosphatidylinositol-4,5-bisphosphate (PI(4,5)P_2_). This assay was validated by confirming the separation of NBD-PC and NBD-PA using HPLC (Fig. S1), verifying that the fluorescence detected from these lipids increased linearly with their concentration in a standard curve (Fig. S2), finding concentrations of PLD1’s C-terminal catalytic domain (PLD1^cat^) and an internal truncation of PLD1’s catalytic domain lacking the disordered loop (PLD1^catΔloop^) that are within the linear range of their activity (Fig. S3), and finding the linear range of PLD1^cat^ with respect to time (Fig. S4).

### ARL11 activation of PLD1 requires the catalytic domain loop

**Figure 1:**
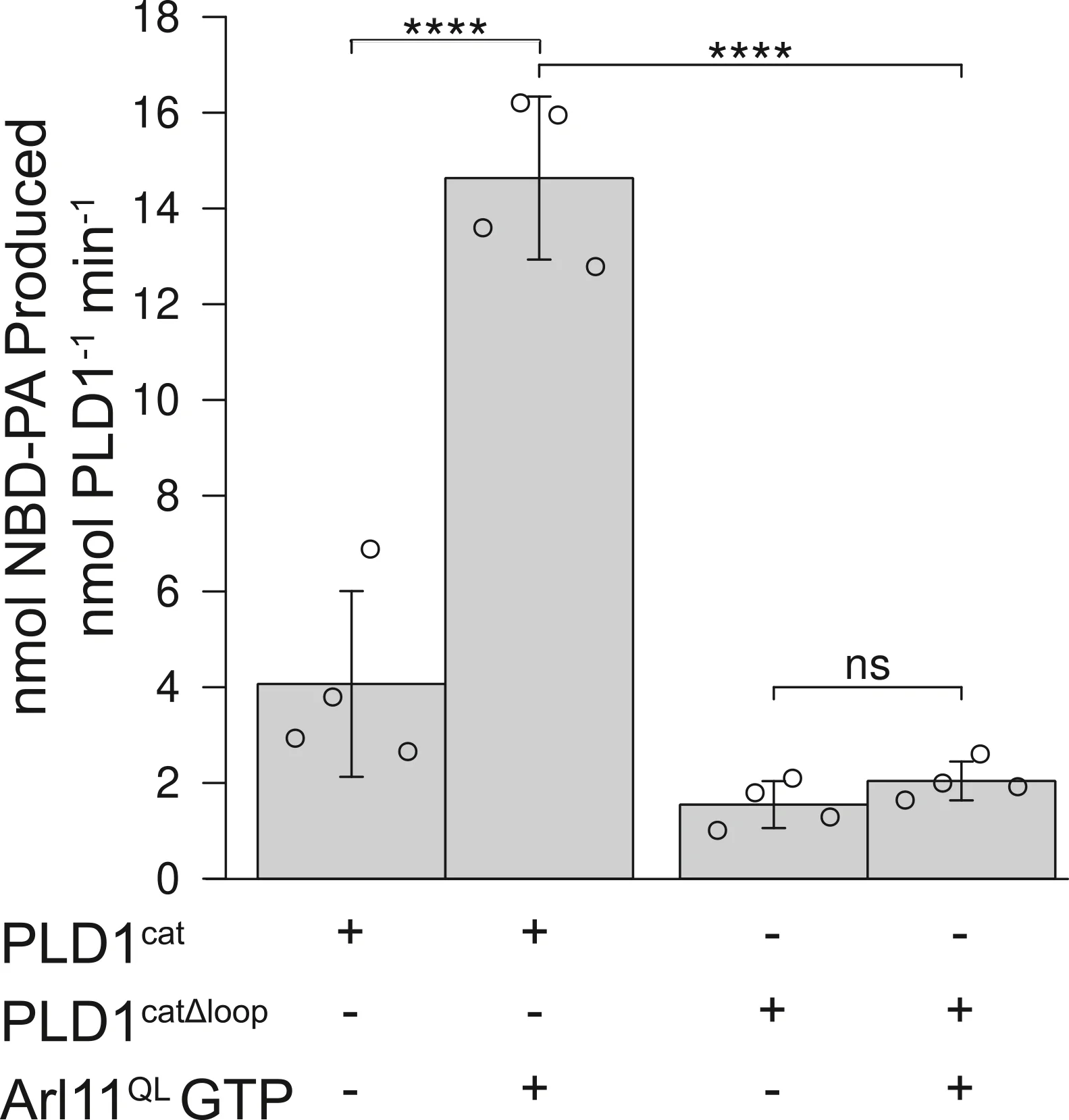
**PLD1 Stimulation by ARL11^QL^ Requires the Catalytic Domain Loop.***In vitro* PLD activity assay in which the activity of PLD1^cat^ and PLD1^catΔloop^ are tested in the presence and absence of GTP-loaded ARL11^QL^. Each datapoint represents the average of two technical replicates, each bar’s value is the average of 4 experimental replicates, and the error bars represent the standard deviation of the 4 experimental replicates. Pairwise significance was determined by a Tukey’s honest significance test following a one-way ANOVA. The *p* value at a 95% confidence interval are reported as asterisks, where “ns” indicates *p* ≥ 0.05, and “∗∗∗∗” indicates 0.0001>*p*.

Next, we set out to test whether we could replicate the stimulation of PLD1 by ARL11, and whether this stimulation could be abolished by abrogation of PLD1’s disordered loop. To do so, the activity of PLD1^cat^ and PLD1^catΔloop^ were measured with and without exposure to GTP-loaded ARL11 (Fig. 1). For this assay, and all subsequent activity assays, we used the Q67L mutant of ARL11 (Arl11^QL^) because it has a drastically reduced rate of GTP hydrolysis and would therefore keep GTP loaded into its nucleotide-binding site (42).

In agreement with prior results, we found that PLD1^cat^ saw an approximately 4-fold increase in activity, but that PLD1^catΔloop^ is completely insensitive to GTP-loaded ARL11^QL^ activation. This increase in activity could not be reproduced by GTP-loaded ARL11^QL^ in the absence of PLD1^cat^ (Fig. S5). Taken together, we concluded that ARL11 stimulated PLD1’s catalytic activity in liposomes, to similar levels as previously observed in Triton X-100 mixed micelles (42).

### ARL11 activation of PLD1 requires GTP-loading

Having established a system that can replicate the prior *in vitro* findings for PLD1 and ARL11’s interaction, our next line of inquiry was to test the dependence of this activation on the nucleotide loaded into ARL11. Although the paradigm of GTPase functionality suggests that ARL11 should only be able to interact with downstream effectors while GTP-loaded and should possess no signaling effects when GDP-loaded (45, 46, 47), it had not yet been experimentally determined whether ARL11 could still stimulate PLD1’s activity while GDP-loaded. To test this, we measured the activity of PLD1^cat^ alone, and while exposed to ARL11^QL^ either loaded with GDP or GTP.

**Figure 2:**
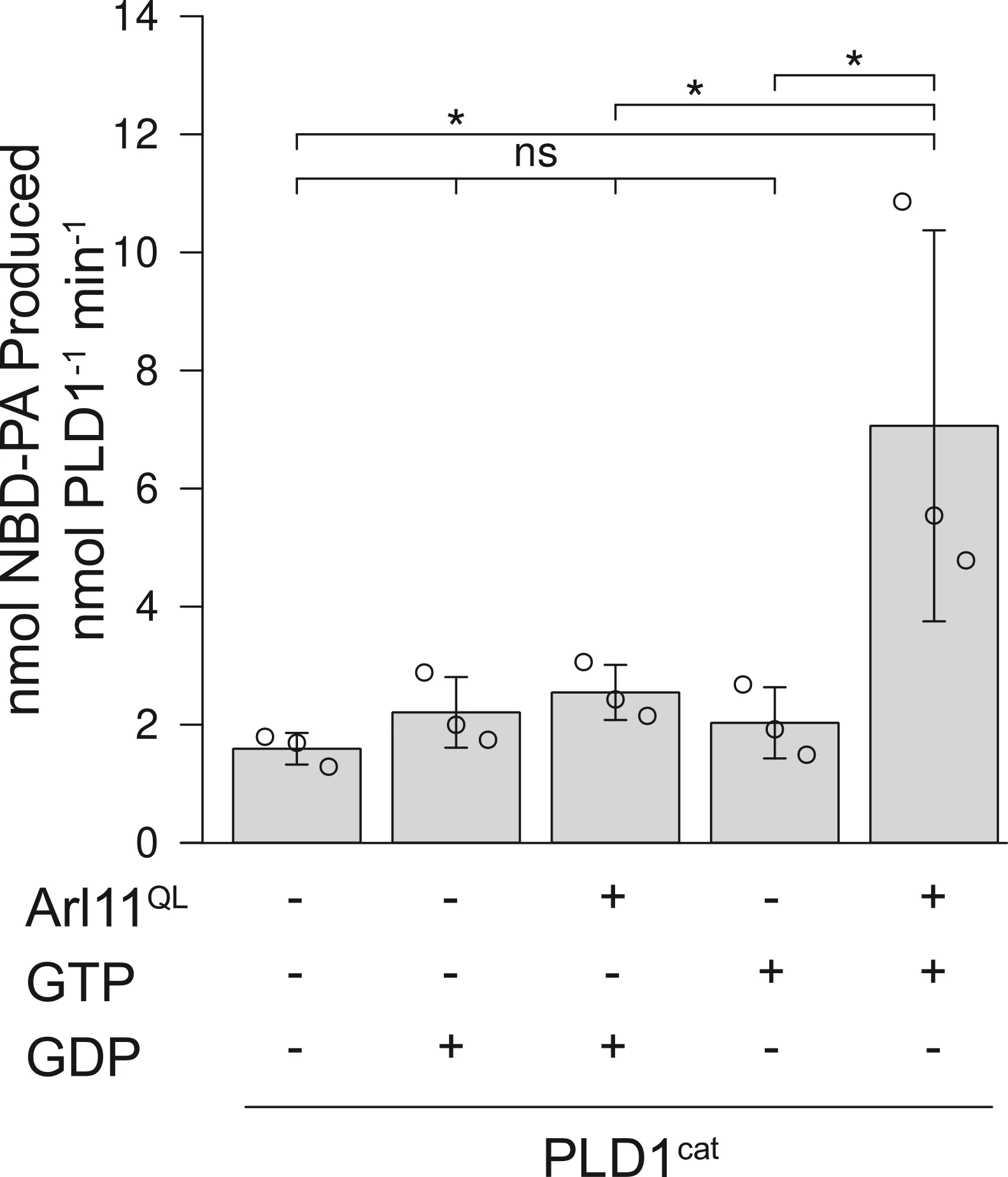
**PLD1^cat^ Stimulation by ARL11^QL^ Requires GTP-Loading.** Effect of nucleotide loading on PLD1^cat^ activity *In vitro.* ARL11^QL^ is loaded with either GTP or GDP, and is introduced to PLD1^cat^ alongside its nucleotide exchange solution, denoted as “GTP” or “GDP”. Each data point represents the average of two technical replicates; each bar’s value is the average of 3 experimental replicates, and the error bars represent the standard deviation of the 3 experimental replicates. Pairwise significance was determined by a Tukey’s honest significance test following a one-way ANOVA. The *p* value at a 95% confidence interval are reported as asterisks, where “ns” indicates *p* ≥ 0.05, and “∗” indicates 0.05>*p* ≥ 0.01.

As expected, ARL11^QL^ did not stimulate PLD1 activity when GDP-loaded (Fig. 2). Thus, ARL11 follows the paradigm of GTPase functionality: requiring bound GTP to stimulate downstream effectors and losing this capability when bound to GDP (45, 46, 47). Although small increases of PLD1^cat^ activity were observed when nucleotide exchange reactions lacking ARL11^QL^ were introduced, this negligible activation by the nucleotide exchange solution was not significant. This confirms that the interaction with GTP-loaded ARL11^QL^, and not GTP alone, is responsible for the observed stimulation of PLD1^cat^.

### AlphaFold 3 prediction of the PLD1-ARL11 complex

**Figure 3:**
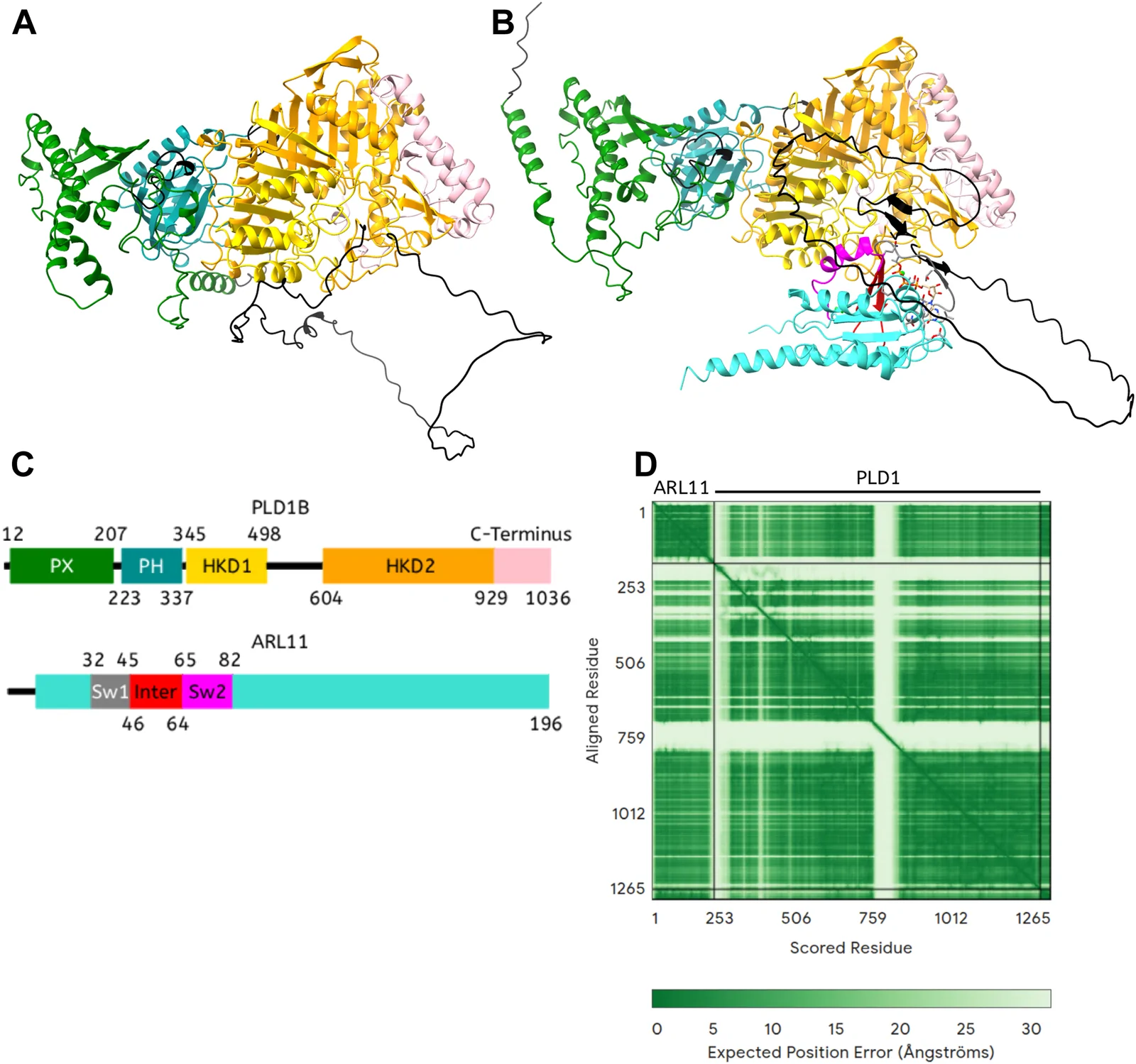
**AlphaFold 3 Structure Predictions of PLD1 Alone and in Complex with ARL11.***A*, structure of PLD1B as predicted by AlphaFold 3 (44) and viewed with ChimeraX (74). Each residue has been colored according to the domain it belongs to, as shown in Figure 3*C*. *B*, structure of the complex between PLD1B and ARL11, including Mg^2+^ (*bright green*) and GTP (*sticks*), as predicted by AlphaFold 3 (44) and viewed with ChimeraX (74). The amino acids of each protein have been colored according to the domain they belong to and their depiction in Figure 3*C*. The PLD1 catalytic domain loop (*black*) is predicted to form ordered secondary structure at its N- and C-terminal ends upon complex formation with ARL11. *C*, the domain architecture of PLD1 and ARL11 are shown. Ordered domains are represented by colored *boxes*, and disordered regions are indicated by thick *black lines*. The amino acid boundaries of each domain, as approximated by examining each protein’s AlphaFold 3 prediction, are indicated as numbers flanking each domain. The boxes and lines are proportionate to the length of the amino acids that they indicate, but the two sequences are not scaled relative to each other. “Sw1”, “Inter”, and “Sw2” are abbreviations of the Switch 1, Interswitch, and Switch 2 regions of ARL11, respectively. *D*, the Predicted Aligned Error (PAE) graph generated by AlphaFold 3 for the predicted complex. High degrees of confidence are indicated in *green* and support the relative orientation of ARL11 and PLD1 in the predicted complex. The low confidence regions near residues 200 and 750, conveyed by a *lighter green* color, correspond to the PLD1 N-terminus and most of its disordered loop, respectively.

To investigate the structural details of this interaction, we used AlphaFold 3 (44) to predict the structure of PLD1 alone (Fig. 3*A*), and in complex with ARL11 in the presence of GTP and a Mg^2+^ ion, which is required by small GTPases to coordinate the binding of GDP or GTP (48) (Fig. 3*B*). The resulting predicted complex between PLD1 and ARL11 had a high degree of confidence according to AlphaFold 3’s predicted aligned error (PAE) for each residue pair, except for the disordered regions of each protein (Fig. 3, *C* and *D*). In contrast, AlphaFold 3 did not predict a confident complex between PLD2 and ARL11 (Fig. S6), consistent with PLD2 having only a minimal loop that has limited homology to PLD1’s loop (49), supporting the accuracy of the predicted complex.

The PLD1-ARL11 complex predicted ARL11 to interface with parts of PLD1’s HKD1-HKD2 catalytic core and loop region. Consistent with an allosteric activation mechanism, the ARL11 binding site was adjacent to the PLD1 active site pocket and near the predicted membrane interface. Interestingly, PLD1’s loop, which is predicted to be entirely disordered in the absence of ARL11 (Fig. 3*A*), forms small regions of secondary structure that are directly involved in complex formation with ARL11 (Fig. 3*B*). However, only the N- and C-terminal edges of PLD1’s loop, consisting of amino acids 498 to 504 and 590 to 603, are predicted to be involved in this interaction. This provides a rational basis for why deletion of the entire loop, consisting of residues 501 to 604, eliminates ARL11 activation but suggests that most of the loop is dispensable for ARL11 stimulation of PLD1 activity.

### The N- and C-terminal ends of PLD1’s disordered loop are predicted to interact with ARL11

**Figure 4:**
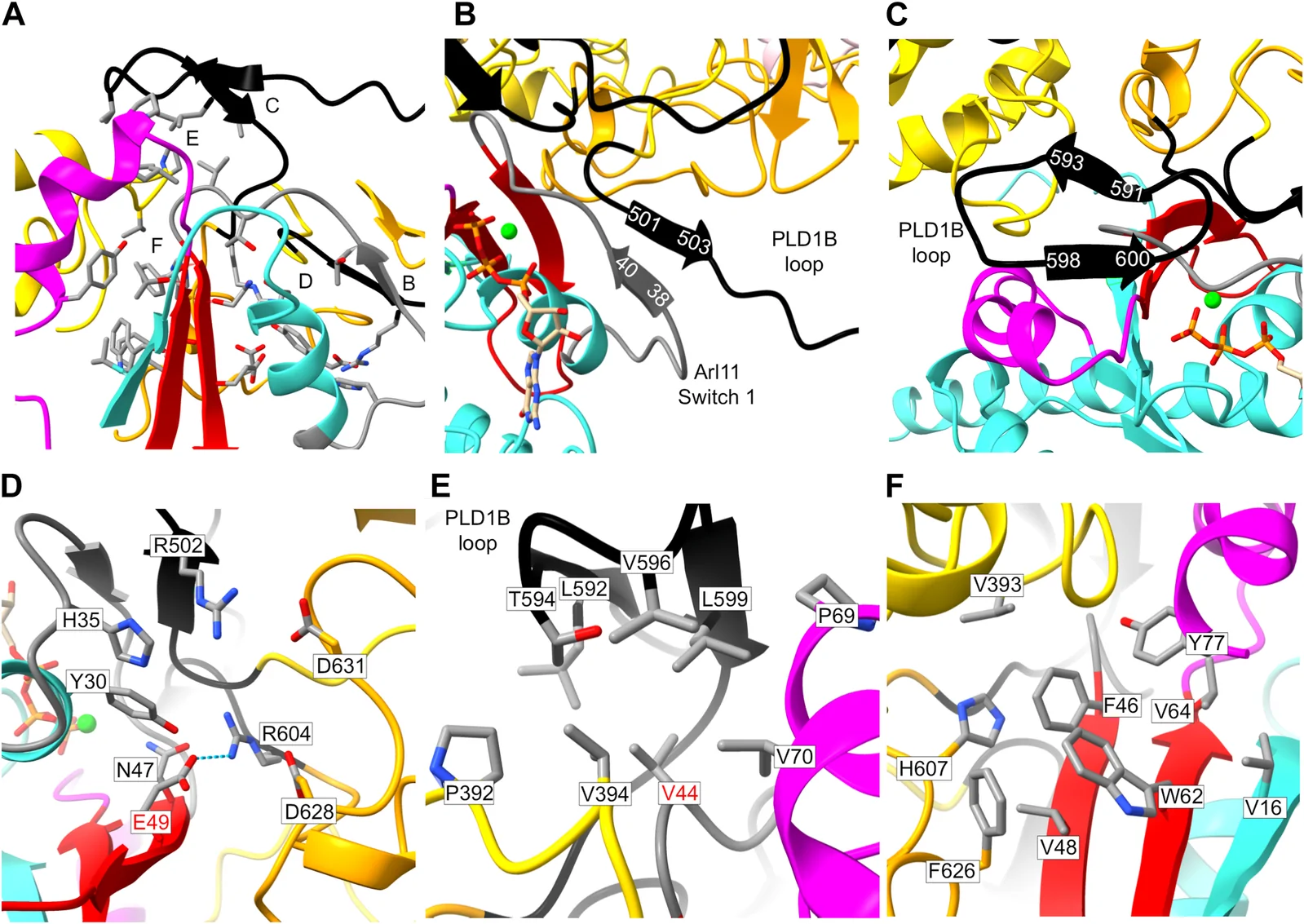
**Features of the PLD1-ARL11 Complex Predicted by AlphaFold 3.***A*, view of the PLD1-ARL11 interface from the side of ARL11. Much of ARL11 in the foreground and PLD1 in the background have been hidden to allow viewing of the interface between them. The features depicted in panels *B–F* are labelled. *B*, AlphaFold 3 predicts an intermolecular 2-stranded beta sheet (*black* and *grey arrows*) to form between the switch 1 region of ARL11 (*grey strand*) and the N-terminal end of a now ordered region within PLD1’s catalytic domain loop (*black strand*). Amino acids at the edges of each protein’s beta strands that form the 2-stranded beta sheet are numbered. GTP is shown as sticks. The Mg^2+^ ion is shown as a *green sphere*. *C*, beta hairpin (*black*) predicted by AlphaFold 3 to form from the C-terminal edge of the PLD1’s catalytic domain loop upon interacting with ARL11. Amino acids at the edges of each beta strand have been numbered. The visible domains of each protein are labeled. GTP is shown as sticks. The Mg^2+^ ion is shown as a *green sphere*. *D*, Polar interface between PLD1 and ARL11 within the predicted complex. The sidechains of amino acids which prompted us to designate this interface as polar are shown and labeled. The visible domains of each protein are labeled. Hydrogen bonds are depicted as dashed *blue lines*. E49 within ARL11’s switch 1 region is denoted in *red* because it was selected for mutagenesis. *E*, hydrophobic interface between PLD1 and ARL11 within their predicted complex. The sidechains of amino acids which prompted us to designate this interface as hydrophobic are shown and labeled. The visible domains of each protein are labeled. V44 within ARL11’s switch 1 region is denoted in *red* because it was selected for mutagenesis. *F*, aromatic interface between PLD1 and ARL11 within their predicted complex. The sidechains of amino acids which prompted us to designate this interface as aromatic are shown and labeled. The visible domains of each protein are labeled. This figure was made in ChimeraX (74) by visualizing structure predictions generated by AlphaFold 3 (44).

Several interactions are predicted to form between PLD1 and ARL11 within their complex (Fig. 4*A*).The regions of ARL11 involved in binding PLD1 comprises ARL11’s switch 1, interswitch, and switch 2 regions (48, 50). A short stretch of residues (residues 500–502) at the N-terminal edge of PLD1’s catalytic domain are predicted to form an anti-parallel intermolecular 2-stranded beta sheet with residues 38 to 40 in ARL11, which comprise part of its switch 1 region (Fig. 4*B*). The C-terminal side of PLD1’s disordered loop is predicted to become ordered, forming a beta hairpin from residues 591 to 600. This putative beta hairpin, much like the predicted secondary structure at the N-terminal side of PLD1’s disordered loop, is predicted to interact with residues contributed by PLD1’s HKD1 domain, and ARL11’s interswitch and switch 2 regions (Fig. 4*C*).

### Three interfaces are predicted to mediate the ARL11-PLD1 interaction

When examining the interactions formed by the surface of ARL11 against PLD1, we categorized these interactions into three interfaces: the polar interface, hydrophobic interface, and aromatic interface, each being named according to the properties of the amino acids that comprise them. The polar interface involves a series of salt bridge interactions between residues in PLD1’s HKD2 domain and the N-terminal side of its loop, with ARL11’s interswitch region, the switch 1 region, and the helix preceding it (Fig. 4*D*). Of note, E49 in ARL11 forms a salt bridge with R604 of PLD1 at the center of the polar interface (Fig. 4*E*). The hydrophobic interface is comprised of residues from the interswitch and switch 2 regions of ARL11, and residues from the HKD1 and C-terminal end of the disordered loop of PLD1. The latter region forms a beta hairpin cap over the former interactors, potentially enabling the formation of this hydrophobic pocket, which would otherwise be exposed to the cytosol (Fig. 4*E*). Finally, the aromatic interface is formed by interactions between ARL11’s interswitch and switch 2 regions and with both HKD domains within the catalytic domain of PLD1 (Fig. 4*F*). The double beta strands of ARL11’s interswitch region that participate in forming the aromatic interface are also involved in forming the polar interface, which is located on the other side of these beta strands.

### Point mutations validate the predicted ARL11-PLD1 interface

**Figure 5:**
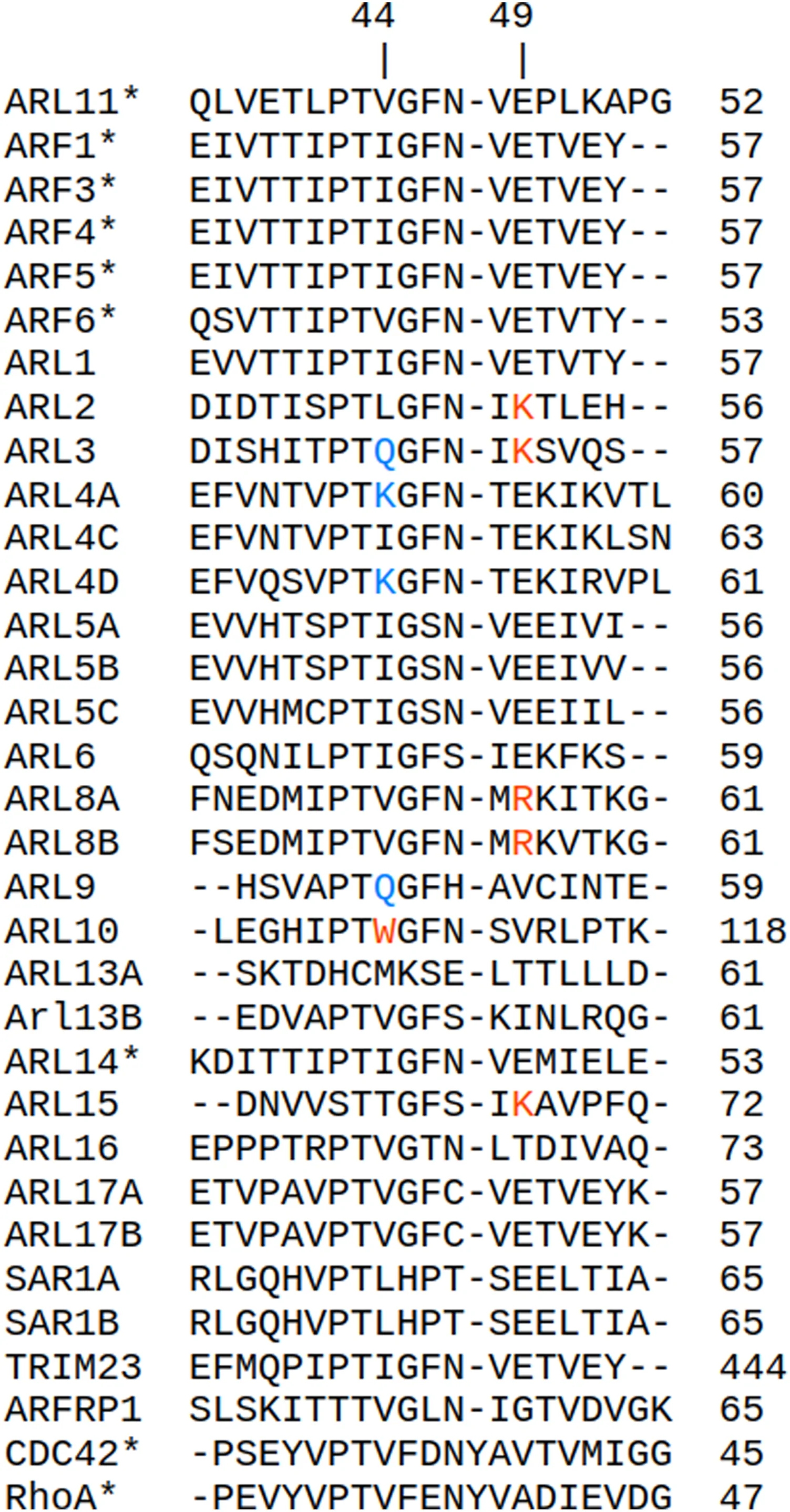
**Sequence Alignment of the ARF Family of Small GTPases and two Additional PLD1 Activators.** Sequence alignment of the primary amino acid sequences of every member of the ARF family of small GTPases, CDC42, and RhoA. Proteins which reportedly stimulate PLD1 are marked with an asterisk. Positions V44 and E49 in ARL11 are indicated above the alignment. Residues marked in *red* match one of the mutations that we introduced into ARL11. Residues marked in *blue* indicate that homologs of ARL11 have charged amino acids in a position homologous to position 44 in ARL11. The numbers to the *right* of the alignment indicate the amino acid position at the far *right* of each sequence. The sequence alignment was conducted using NCBI’s COBALT (78) multiple sequence alignment server and the canonical amino acid sequence listed for each protein by Uniprot (73).

To validate the predicted interactions, we designed a series of point mutations in ARL11 intended to disrupt the predicted complex. To avoid mutations that would disrupt the structure of ARL11 or its ability to bind GTP, we first aligned the amino acid sequence of ARL11 with all 31 members of the ARF GTPase family (41, 45, 46, 51). This allowed us to identify ARL11 residues that were predicted to interact with PLD1 but were not universally conserved in other ARF-family GTPase members (Fig. 5). We reasoned these residues were ideal to mutate in ARL11, as they undergo endogenous variation in other functional GTPases and are therefore unlikely to affect protein folding and GTP binding. Based on this analysis, we chose V44 in ARL11’s switch 1 region and E49 in ARL11’s interswitch region as the sites for mutagenesis, which are in the predicted hydrophobic and polar interfaces, respectively.

To disrupt the polar interface, we generated E49R (ARL11^ER^) and E49K (ARL11^EK^) point mutants. Arg and Lys residues were chosen to switch the sidechain charge from the native negatively charged glutamate in ARL11 to positively charged Arg and Lys residues. Notably, both of these mutations can be found within the ARF family. The E49R mutation matches ARL8A and ARL8B, which both possess an arginine residue in the position homologous to E49 within ARL11. Similarly, ARL2, ARL3, and ARL15 possess an endogenous lysine residue at a position homologous to ARL11’s E49, mirroring our E49K mutant.

To disrupt the hydrophobic interface, we generated V44R (ARL11^VR^) and V44W (ARL11^VW^) point mutants. Arginine was chosen to disrupt the predicted hydrophobic interface by introducing a charged, polar residue, while tryptophan was chosen for its size so that it could disrupt the hydrophobic interface by steric clashes. Like our ARL11 E49 mutants, precedents for these mutations can be found within the ARF GTPase family. Specifically, ARL4A and ARL4D both possess a lysine residue at a position homologous to V44 in ARL11, suggesting that our introduction of a positively charged residue in our V44R mutant is unlikely to disrupt the structure of ARL11. Correspondingly, the V44W mutant mimics ARL10, which also possesses a tryptophan residue at a position homologous to V44 in ARL11. Further supporting that these point mutations do not interfere with ARL11 folding, all point mutant constructs exhibited size-exclusion profiles comparable to ARL11^QL^ (Fig. S7).

**Figure 6:**
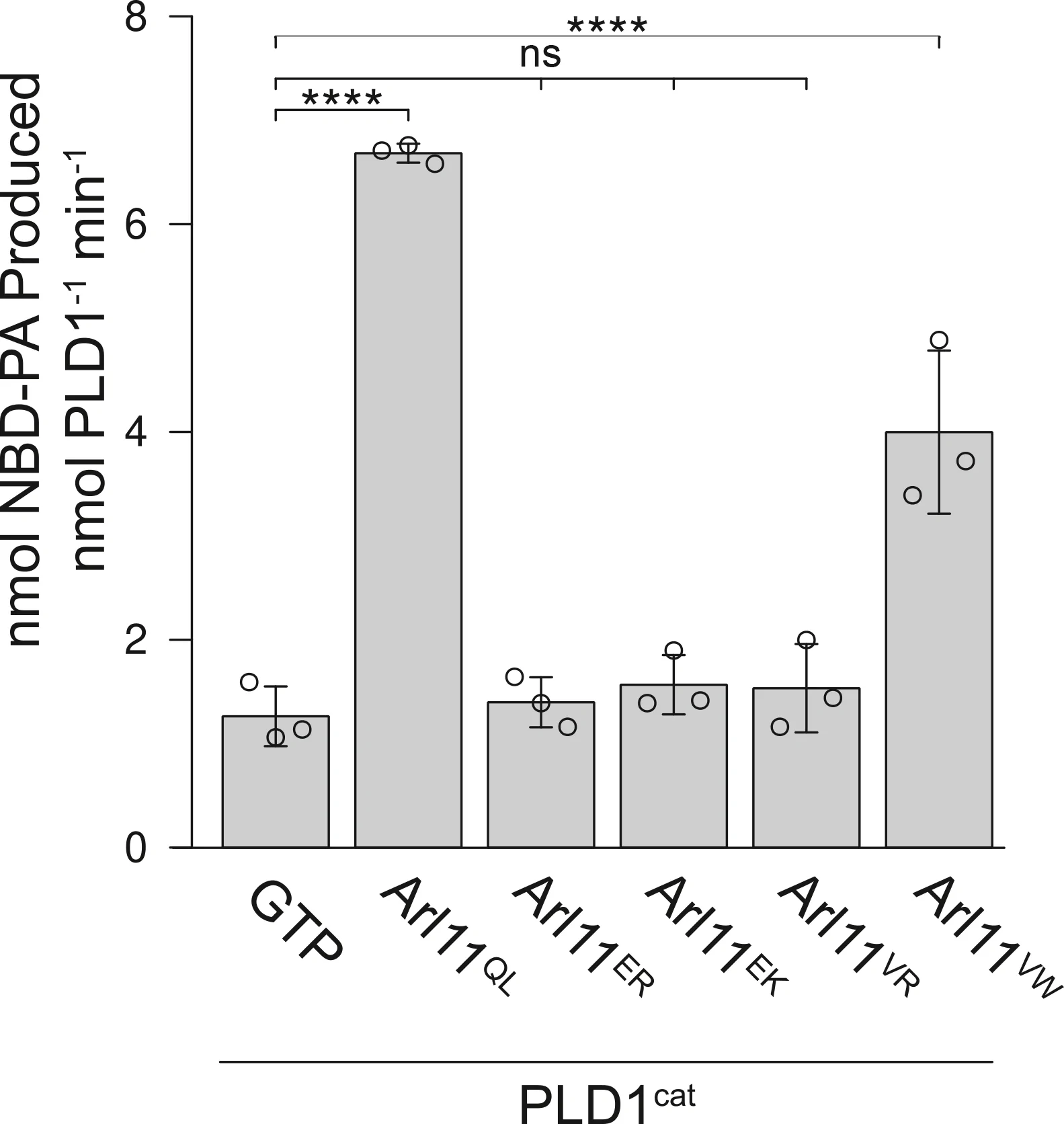
**Point Mutants of ARL11^QL^ Show a Reduced Capacity to Stimulate PLD1^cat^.***In vitro* PLD activity assay where PLD1^cat^ is exposed to GTP-loaded ARL11^QL^, ARL11^E49R^, ARL11^E49K^, ARL11^V44R^, and ARL11^V44W^. The nucleotide exchange solution used to load GTP into the ARL proteins was used as a negative control. Each data point represents the average of two technical replicates; each bar’s value is the average of 3 experimental replicates, and the error bars represent the standard deviation of the 3 experimental replicates. Pairwise significance was determined by a Tukey’s honest significance test following a one-way ANOVA. The *p* value at a 95% confidence interval are reported as asterisks, where “ns” indicates *p* ≥ 0.05, and “∗∗∗∗” indicates 0.0001>*p*.

We then tested the ability of these ARL11 point mutants to activate PLD1. We found that both the E49R and E49K mutations completely abrogated ARL11’s stimulation of PLD1^cat^ (Fig. 6), demonstrating that this glutamate residue, and the interswitch region of ARL11 in general, which was predicted to form contacts with PLD1, are necessary to stimulate PLD1. Similarly, disrupting the putative hydrophobic interface of the ARL11-PLD1 complex by introducing the V44R to ARL11 also abolished stimulation of PLD1^cat^. Interestingly, the V44W mutation diminished the stimulation of PLD1^cat^ by half but did not eliminate ARL11-mediated PLD1 activation. We speculate that the tryptophan’s hydrophobicity allows it to still interact favorably with the hydrophobic interface of PLD1, but its larger size leads to a partial disruption of the interface. Regardless, the two V44 mutations demonstrate that the hydrophobic interface is essential for ARL11 to fully stimulate PLD1^cat^, and the two E49 mutations demonstrated the same for the polar interface. Taken together, these results support the interactions of ARL11 with PLD1 within their predicted complex.

### The majority of PLD1’s loop is dispensible for ARL11 stimulation

**Figure 7:**
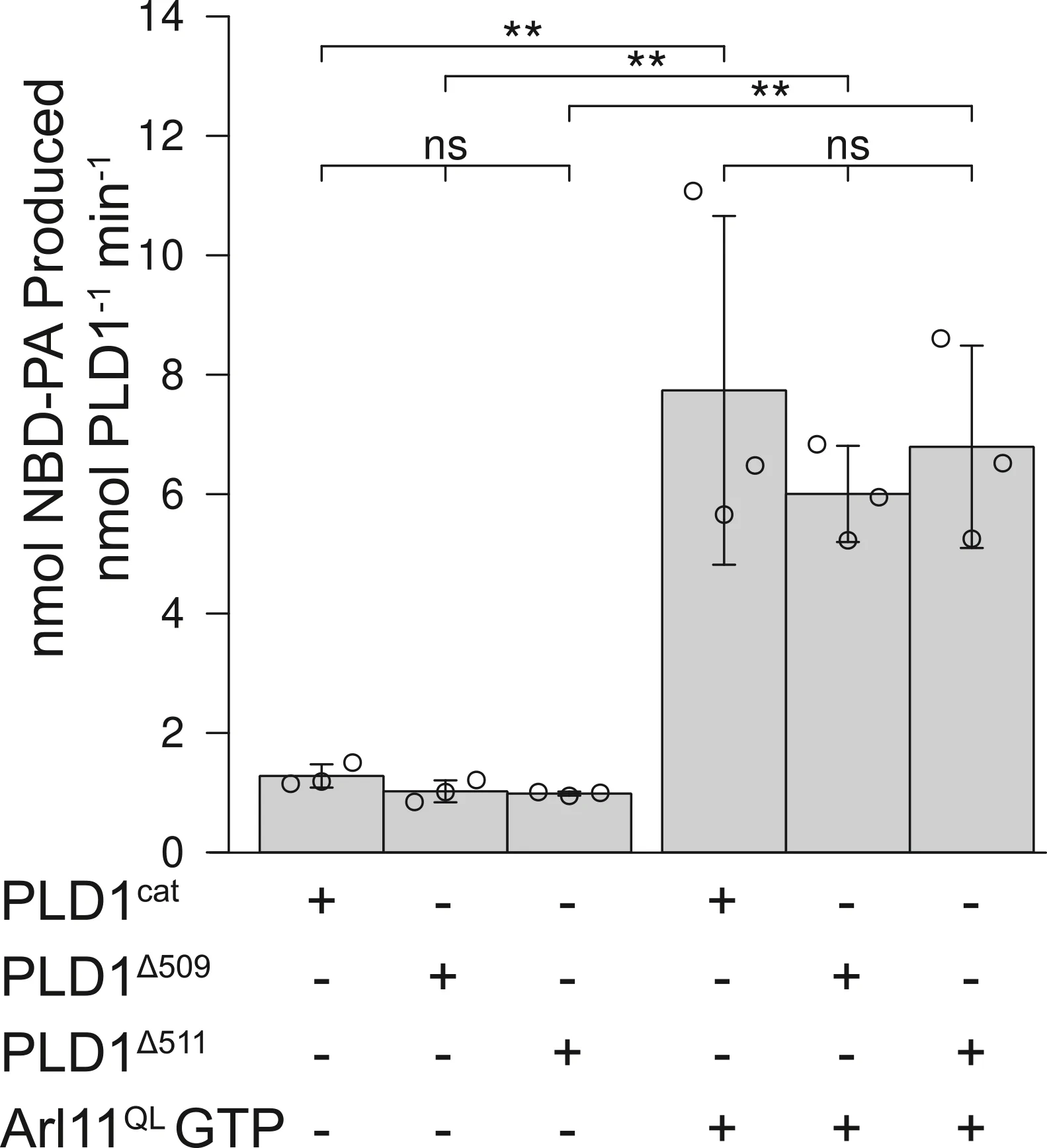
**The Majority of the PLD1 Catalytic Loop is Dispensable for ARL11 Stimulation.***In vitro* PLD activity assay where the activity of PLD1^cat^, PLD1^Δ509^, and PLD1^Δ511^ are either tested alone, or while exposed to GTP-loaded ARL11^QL^. Each data point represents the average of two technical replicates, each bar’s value is the average of 3 experimental replicates, and the error bars represent the standard deviation of the 3 experimental replicates. Pairwise significance was determined by a Tukey’s honest significance test following a one-way ANOVA. The *p* value at a 95% confidence interval are reported as asterisks, where “ns” indicates *p* ≥ 0.05, and “∗∗” indicates 0.01>*p* ≥ 0.001.

Having identified at least two ARL11 residues that are critical for complexation with and stimulation of PLD1, we turned our attention to testing whether the majority of PLD1’s disordered loop between its N- and C-terminal residues is dispensable for activation by ARL11. We generated two deletion mutants of PLD1^cat^: PLD1^cat^ Δ509 to 585 (PLD1^Δ509^) and PLD1^cat^ Δ511 to 581 (PLD1^Δ511^), which both retained the residues at the N- and C-terminal edges of the loop that are predicted to mediate interaction with ARL11. Upon comparing their activity to PLD1^cat^, we found no significant difference in the degree of stimulation by ARL11^QL^ (Fig. 7). Thus, we concluded that the majority of PLD1’s disordered loop is dispensable for its stimulation by ARL11, and that a truncated loop consisting only of residues 501 to 508 and 586 to 604 is sufficient for stimulation by ARL11.

## Discussion

While the activity of human PLD1 has been studied for decades (4), ARL11 was only recently found to stimulate its activity, and, alongside ARL14, remains one of the least studied members of the ARF family of small GTPases (42). Here, we present a series of biochemical studies that define the key features of this process. This includes the GTP dependence for ARL11 activation, predicting the structural interface for the interaction between PLD1 and ARL11, and revealing both residues within ARL11 that are necessary, and limited regions of the PLD1 specific loop that are sufficient, for ARL11 activation of PLD1. Because ARL14 and ARL11 have higher sequence homology to each other than any other small GTPase of the ARF family (42), and because ARL14 has already been shown to colocalize with and stimulate PLD1 (42), we propose that the interaction details of ARL11 and PLD1 elucidated here also apply to ARL14’s interaction with PLD1.

For our biochemical studies, we devised a novel means of measuring PLD1 activity. NBD-PC was delivered in liposomes as the PLD1 substrate, and HPLC was used to resolve and quantitate the substrate and NBD-PA product. Previously, NBD-PC had been introduced in micelles (52, 51), which are more disimilar to PLD1’s native membrane environment than liposomes, and had been resolved by thin layer chromatography (TLC) (53), which cannot run samples as quickly as HPLC and cannot be quantitated as accurately. Although both NBD-PC (54, 55) and HPLC (4) have been employed to measure PLD1 activity before, this study is the first instance of their co-implementation to our knowledge.

Although it is clear that ARL11 activates PLD1, the level of activation remains undefined and may exceed the 4- to 6-fold activation we observed here. This is due to our experimental conditions, which were optimized to allow for detection of unstimulated PLD1 activity in the linear range. However, in many of the assays in which PLD1^cat^ was stimulated by GTP-loaded Arl11^QL^, roughly 50% of the original NBD-PC substrate had been converted to product (Fig. S8). Given only ∼50% of the total NBD-PC substrate would be available to PLD1^cat^ on the outer leaflet of liposomal membranes, this suggests that the PLD reaction had gone to completion. Thus, we are likely underestimating the extent to which Arl11^QL^ can stimulate PLD1 activity. This is in spite of the fact that the liposome substrate contains 50 mol% PC, which far exceeds the 23 mol% PC estimated to be within the inner leaflet of the plasma membrane of human cells (56). Therefore, stimulation of PLD1 by ARL11 *in vivo* may generate extremely high local concentrations of PA through rapid conversion of PC to PA by activated PLD1.

The work presented here advances our understanding for the stimulation of PLD1 by ARL11, as it defines the interface and key residues involved in this protein-protein interaction. However, the mechanistic basis for PLD1 activation by ARL11, and other GTPases remains unclear. We propose two plausible mechanisms, which are not mutually exclusive and could work in tandem to activate PLD1: ARL11 may produce allosteric structural changes in PLD1, and it may promote membrane interaction of PLD1’s catalytic domain. For allosteric activation, binding of ARL11 to the periphery of PLD1’s HKD1-HKD2 catalytic domain could induce structural changes in the active site of the PLD1 catalytic domain that would increase catalysis, as has been found for other GTPase activators of PLD1 (57).

In contrast to this conventional activation mechanism, the possibility that ARL11 might stimulate PLD1 by promoting membrane association is more complicated, given that PLD1 is constitutively membrane-associated (58, 59, 60). However, PLD1 membrane binding is largely mediated by its N-terminal tandem Phox Homology (PX) and Pleckstrin Homology (PH) domains (58), with only a phosphatidylinositol 4,5-bisphosphate (PI(4,5)P_2_) polybasic binding pocket within the catalytic domain known to be involved in membrane association (58). Therefore, it is plausible that PLD1’s catalytic domain may normally be only loosely associated with the membrane, relying on the PX and PH domains to prevent the enzyme from fully dissociating, and that ARL11 may stimulate PLD1’s activity by binding to and anchoring the catalytic domain at membranes to increase catalysis. Lending credence to this hypothesis, AlphaFold 3 predicts ARL11 to associate with PLD1 in such a way that it would be resting on the membrane interface. Furthermore, ARL11 possesses an N-terminal MGSXXS motif (Fig. S9) which may mark it for myristoylation by N-myristoyltransferases. This suggests ARL11 may associate with membranes using this N-terminal lipidation while bound to GTP, as is typical of ARF GTPases (45), including activators of PLD1 such as ARF1 and ARF6 (Fig. S9). Thus, ARF GTPases like ARL11 may anchor both themselves and the PLD1 catalytic domain to membranes in order to promote PLD1 catalysis. Future structural studies that capture PLD1 bound to ARL11 are needed to answer these mechanistic questions.

In addition to stimulating PLD1’s activity, it remains possible that complexation between ARL11 and PLD1 may inhibit the hydrolysis and release of ARL11’s bound GTP, meaning that PLD1 would serve as a guanine nudelotide dissociation inhibitor (GDI) for ARL11. In GTPases, the switch 1 region is responsible for either encaging the enzyme’s bound nucleotide, or forming a third strand in a beta sheet alongside two beta strands of the interswitch region to allow the GTPases’ bound nucleotide to dissociate (61). In the predicted complex, ARL11’s switch 1 region traps the bound GTP while simultaneously interacting with PLD1, which suggests that complexation between the two enzymes may discourage ARL11 from releasing its bound nucleotide. Further supporting this idea, Q67, which is a key residue for ARL11’s catalytic activity and is substituted to a Leu residue in ARL11^QL^, is predicted to form two hydrogen bonds with the backbone amide and carbonyl oxygen atom of residue 600 in PLD1’s disordered loop, which could reduce the ability of Q67 to perform its canonical function of abstracting hydrogen from a water molecule to catalyze hydrolysis of the gamma phosphate of GTP (62).

One avenue of experimentation that remains unexplored is whether ARL11’s stimulation of PLD1 is mutually exclusive or additive with stimulation by ARF1 or RhoA. It has been demonstrated before that exposure to ARF1 and RhoA has a synergistic effect on PLD1 stimulation, whereas ARF1 and ARF6 compete for the same binding site and do not additively stimulate PLD1 together (28). Because AlphaFold 3 predicts that ARL11 and ARL14 bind to the same surface of PLD1 as ARF1 and ARF6 (Fig. S10), we speculate that stimulation by ARL11, ARL14, ARF1, and ARF6 would not be additive, while stimulation with ARL11 or ARL14 and RhoA would be.

Because of its essential role in enabling small ARF-family GTPases like ARL11 to stimulate the activity of PLD1, we propose that this loop should be named the ARF-associating loop. Previously, this loop within PLD1’s catalytic domain was posited to be disordered and autoinhibitory (35, 63). It was thought to be responsible for the lower basal enzymatic activity of PLD1 as compared to PLD2 (63), which possesses an attenuated loop with limited homology to PLD1’s loop and higher basal activity (63). However, removal of the loop from PLD1 did not significantly increase its basal activity in our hands (Fig. 1), as would be anticipated for an autoinhibitory loop. Instead, the sensitivity of PLD1 to ARL11 stimulation was abolished. Our data suggest that the function of this loop is not to reduce the activity of PLD1, but to form ordered structures in the presence of some ARF GTPases and mediate their interaction with PLD1. Thus, we believe that “PLD1 disordered loop” is no longer an appropriate name for this region of PLD1.

It should be noted that PLD1 possesses multiple isoforms due to alternative splicing (28), and its A and B isoforms differ in the length of their ARF-associating loops. The A isoform is longer by 38 residues, which would be present between residues 585 and 586 in the B isoform: before the C-terminal region of the ARF-associating loop predicted to interact with ARL11 by AlphaFold 3. Because of this, and because we were able to demonstrate activation of PLD1 by ARL11 using the shorter B isoform lacking these 38 residues, we believe that the A isoform of PLD1 would not respond differently than the B isoform to ARL11 interaction.

AlphaFold has been proposed as a tool to discover protein-protein interactions (44, 64, 65). We tested this supposition using PLD1 and the ARF/ARL family, whose members have been experimentally screened in cells for interaction with PLD1 (42, 66). In this analysis, we found that AlphaFold 3 reported high PAE confidence scores for known, experimentally verified interactors with no false negative predictions. This is also reflected in pTM and IpTM (50, 52) scores of at least 0.8 for each of the known activators: ARL11, ARL14, ARF1, ARF3, ARF4, ARF5, and ARF6 (28, 49, 50, 52). Using these values as benchmarks to assess the reliability of the complexes predicted for the other ARF family members, ARL4C, ARL5A, and ARL5B were predicted to bind to PLD1 with similar levels of confidence (Fig. S11). This predicts that these small GTPases may be potential activators of PLD1. However, these GTPases were not detected as PLD1 binding partners by TurboID (66) or Mini TurboID (42), which had identified ARL11 and ARL14 as novel PLD1 interactors. While we cannot rule out that PLD1 is also activated by other GTPases, this suggests that experimental verification should be mandatory for any high-confidence protein-protein interaction predictions.

The physiological role of ARL11 activation of PLD1 remains unclear. There is contradictory information about the role of ARL11 in cancer (67, 68), and none of the heart defect mutations discovered in PLD1 (69) lie at the PLD1-ARL11 protein-protein interface with ARL11, so ARL11 is unlikely to play a role in PLD1’s contribution to heart defects. However, there are several reports of ARL11 being involved in macrophage activation (70), immune responses (71), and phagocytosis (42). The phagocytosis activation has been confirmed to be transduced through PLD1 (42), but it remains unknown whether ARL11 transduces its effects on macrophage activation and immune response through activation of PLD1, and future studies are needed to verify this.

## Experimental procedures

### Plasmids

PLD1^cat^ was generated as previously described (52), with residues 330 to 1036 from human PLD1B (28), corresponding to the catalytic domain, cloned into the pFastBac HtB plasmid (Gibco, Life Technologies) using SfoI and NotI restriction sites. This construct has an N-terminal 6-His tag with a Tobacco Etch Virus (TEV) cleavage site. PLD1^catΔloop^ was cloned using overlap extension to delete residues 501 to 604 from PLD1^cat^ and create a version of PLD1^cat^ lacking the disordered loop of its catalytic domain. To generate versions of PLD1 with a truncated, but not fully removed, disordered loop, divergent PCR was used to create linear amplicons lacking the deleted regions. The deletions were then verified by sequencing, resulting in the constructs PLD1B 330 to 1036 Δ509 to 585 (PLD1^Δ509^) and PLD1B 330 to 1036 Δ511 to 581 in pFastBac HtB (PLD1^Δ511^).

Ligation independent cloning (71) was used to clone human ARL11 Q67L into the plasmid ppSUMO, which is a derivative of pET28a that has a SUMO protein inserted between the NdeI and BamHI sites. ARL11 Q67L expressed from these plasmids had an N-terminal 6xHis-SUMO tag, and is designated as ARL11^QL^. Divergent PCR using ARL11 Q67L in ppSUMO as a template was used to generate ARL11 Q67L V44R (Arl11^V44R^), ARL11 Q67L V44W (Arl11^V44W^), ARL11 Q67L E49R (Arl11^E49R^) and ARL11 Q67L E49K (Arl11^E49K^) and the presence of each point mutation was verified by sequencing. The DNA and amino acid sequences for all constructs are included in the supplemental materials.

### ARL protein expression

Arl11^QL^, ARL11^V44R^, ARL11^V44W^, ARL11^E49K^, and ARL11^E49R^ were transformed into BL21-CodonPlus(DE3)-RIPL *E. coli* (Agilent, Catalog #230246), and plated on an LB-agar plate (0.015 g/ml agar, Ref 214530, Difco) containing 50 μg/ml kanamycin. Plated cells were grown overnight at 37 °C, and a single colony from each plate was inoculated in a 30 ml solution of LB with 50 μg/ml kanamycin, and shaken at 37 °C, overnight. 10 ml from each overnight culture was inoculated into a 1 L solution of terrific broth (IB49142, IBI Scientific) with 50 μg/ml kanamycin and 0.4% glycerol (v/v), and 1:10,000 silicone emulsion (Catalog No. 02-002-333, J.T. Baker). The inoculated solutions were housed in 2.5 L baffled flasks (ultra yield flask, Thomson), and shaken at 250 RPM in a machine with a 2.54 cm orbital diameter, and at 37 °C. When the optical density at 600 nm reached 1.5 to 2.5 (10 mm path length), 1 ml of 60 mg/ml IPTG (Cat# I2481c100, GoldBio) was added to each solution, and they were shaken under the same conditions for a further 3 h. After 3 h, each 1 L of solution was centrifuged at 4500*g* at 4 °C for 20 min. The resulting pellet from each 1 L solution was divided in half and then frozen at −80 °C until needed for protein purification.

### PLD1 bacmid generation and expression

PLD1^cat^, PLD1^catΔloop^, PLD1^Δ509^, and PLD1^Δ511^ were transformed into DH10bac *E. coli* (catalog number 10361012, ThermoFisher Scientific), and plated on LB-agar (0.015 g/ml agar, Ref 214530, Difco) plates containing, 50 μg/ml kanamycin, 7 μg/ml gentamycin, 10 μg/ml tetracycline, 100 μg/ml Xgal (Cat #X4281C, GoldBio), and 40 μg/ml IPTG (Cat #I2481C100, Gold Bio). Plated cells were grown for 48 h at 37 °C, and four white colonies from each plate were restreaked onto LB-agar plates of the same composition as above and grown at 37 °C for 48 h. A single white colony from each of the restreaked plates was used to inoculate a 3 ml solution of LB, 50 μg/ml kanamycin, 7 μg/ml gentamycin, and 10 μg/ml tetracycline, which was then shaken at 250 RPM at 37 °C, overnight. Bacmids were purified from the overnight cultures using isopropanol precipitation.

The resulting bacmids were transfected into *Spodoptera frugiperda* (Sf9) cells (Catalog Number 11496015, Gibco). Briefly, 5 μl of bacmid and 6 μl of cellfectin (Ref 58760, Gibco) reagent were diluted in 200 μl of insect cell medium (sf-900 II SFM, REF 10902-088, Gibco) lacking antibiotics, and incubated at room temperature for 15 min before being diluted with an additional 800 μl of antibiotic-free medium. Each 1 ml transfection mix was then added to the well of a 6-well plate (Cat# CC7682-7506, CytoOne) containing 0.5e6 adherent insect cells. The insect cells were then incubated at room temperature for 5 h, before the transfection mix was washed off and replaced with 2 ml of insect cell medium with 1X antibiotic and antimycotic (REF 15240-062, Gibco). Insect cell medium used in all subsequent steps also contained 1X antibiotic and antimycotic. After 72 h at room temperature, the 2 ml of medium containing viruses was harvested and 1 ml of it was used to infect 1.5 ml of 5.6e6 cells/ml adherent insect cells in a 10 cm plate (130182, ThermoScientific). After 96 h at room temperature, the 10 ml of medium was harvested and 1 ml of it was used to infect 50 ml of 1.7e6 cells/ml insect cells. The infected cells were then shaken in 125 ml baffled flasks (Cat No. BBV125, Fisher Scientific) at 125 RPM in a machine with a 2.54 cm orbital diameter at 27 °C for 72 h. The insect cell medium containing the viruses was harvested by centrifugation at 200*g* for 10 min at room temperature, and designated as the third passage of each virus. Each virus was passaged once more by adding 5 ml of passage 3 to 50 ml of 2.4e6 cells/ml insect cells in insect cell medium, shaking for 72 h as described above, and harvesting by centrifugation as described above. 16 ml of the fourth passage of each virus was then used to infect 800 ml of 1.4e6 cells/ml insect cells in insect cell medium. These larger volumes were shaken in 2 L baffled flasks (Cat No. BBV2000, Fisher Scientific) at 125 RPM in a machine with a 2.54 cm orbital diameter and at 27 °C for 72 h before being spun down at 200*g* for 10 min at room temperature. Finally, the pellets from the centrifugation of the larger volumes were stored at −80 °C until needed for purification.

### ARL protein purification

To purify ARL11^QL^, ARL11^V44R^, ARL11^V44W^, ARL11^E49K^, and ARL11^E49R^, a pellet of frozen RIPL *E. coli* was thawed on ice. A lysis buffer, consisting of 500 mM NaCl, 20 mM Tris at pH 7.5, 5 mM imidazole at pH 7.5, and 1 mM 2-mercaptoethanol was added to the thawed pellet 25 ml at a time, and shaken vigorously to resuspend the pellet. The 100 ml were sonicated on ice using a large tip sonicator (FB705, FisherBrand) set to 85% amplitude, 2 s on, 6 s off, for 40 min. After sonication, the cell lysate was centrifuged at 100,000*g* and 4 °C for 30 min. The target protein was then enriched by dividing the supernatant evenly into two 5 ml Nickel-nitrilotriacetic acid (Ni-NTA) resin (CAT#H-350-100, GoldBio) gravity columns kept cold in a 4 °C room, and pre-equilibrated with 20 ml of lysis buffer. After, each column was washed with 20 ml of the lysis buffer, then 20 ml of a wash buffer(500 mM NaCl, 20 mM Tris at pH 7.5, 60 mM imidazole, 1 mM 2-mercaptoethanol). The target protein was eluted using 10 ml of elution buffer per column, consisting of 150 mM NaCl, 20 mM Tris pH 7.5, 300 mM imidazole, and 1 mM 2-mercaptoethanol.

The 6xHis-SUMO tag was cleaved and separated from the ARL proteins by reverse Ni-NTA as described in the following with all steps carried out at 4 °C. Three desalting columns (7322010, BioRad) were equilibrated with 20 ml of water, then 30 ml of buffer A consisting of 150 mM NaCl, 20 mM Tris pH 7.5, 50 mM imidazole and 10 mM 2-mercaptoethanol. The two 10 ml Ni-NTA column elutions were pooled together, then 3.33 ml was added to each desalting column, and eluted as 1 ml fractions with 10 ml of buffer A. This process was repeated once so that all 20 ml of Ni-NTA column elution was buffer exchanged with buffer A. Next, the purified ARL11 proteins were loaded with GTP to prevent precipitation during overnight cleavage. The ARL11 desalting elutions with the highest protein concentration were pooled until a volume of 12 ml was achieved. 650 μl of 100 mM EDTA was added, and the solution was incubated at room temperature for 30 min. Then, 65 μl of 0.2 M GTP was added and the solution was incubated at room temperature for 1 h. Finally, 130 μl of 100 mM MgCl_2_ was added, and the solution was incubated at room temperature for 15 min (21). Before the GTP-loaded ARL11 was incubated at 4 °C overnight, 100 uL of 1.4 mg/ml ULP-1 (purified from RIPL *E. coli* using pET28a vector) was added to cleave the 6xHis-SUMO tag. The next day, the cleaved 6xHis-SUMO fragment was separated from the GTP-loaded ARL11 by reverse Ni-NTA. A single gravity column containing 5 ml of Ni-NTA beads was equilibrated with 20 ml of buffer A and then the ∼13 ml solution of cleaved 6xHis-SUMO and GTP-loaded ARL11 were poured through the column, and the flowthrough was collected (tag-free ARL11). The flowthrough was then passed over the column an additional two times to ensure that all 6xHis-SUMO domain had been bound.

The tag-free ARL11 was further purified by size exclusion chromatography. The elution was filtered through a 0.45 um filter (Cat No. 13-1001-05, Fisher Scientific) and loaded into a 10 ml superloop before being injected onto a Hiload 26/600 Superdex 75 pg column (Cytiva). The liquid phase consisted of 150 mM NaCl, 20 mM Tris at pH 7.5, 10 mM 2-mercaptoethanol, and 1 mM MgCl_2_. Elution fractions containing the ARL11 protein were then pooled together and concentrated with a series of 5 min, 4000*g* spins in a 10 kDa 15 ml concentrative spin column (Ref UFC901024, MilliporeSigma). The concentration of the protein was monitored by measuring absorbance at 280 nm with a baseline correction of 340 nm and once the volume had reached 1 ml, the concentrate was then aliquoted and flash-frozen in liquid nitrogen before being stored at −80 °C.

### PLD1 protein purification

To purify PLD1^cat^, PLD1^catΔloop^, PLD1^Δ509^, and PLD1^Δ511^, a pellet of frozen insect cells spun down from an 800 ml growth was thawed on ice. A lysis buffer, consisting of 500 mM NaCl, 20 mM Tris at pH 7.5, 5 mM imidazole at pH 7.5, and 1 mM 2-mercaptoethanol was added to the thawed pellet 25 ml at a time, and shaken vigorously to resuspend the pellet. Protease inhibitors were then added to bring the solution to 1 mM PMSF (Cat#P-470-25, GoldBio), 10 μM leupeptin (CAS#103476-89-7, GoldBio), 1 μM aprotinin (CAS# 9087-70-1, GoldBio). 100 ml of resuspended cells was poured into a 250 ml metal cup resting in ice water and sonicated using a large tip sonicator (FB705, FisherBrand) set to 50% amplitude, 2 s on, 6 s off, 10 min. After sonication, the cell lysate was centrifuged at 100,000*g* at 4 °C for 30 min. The target PLD1 protein was then enriched from the supernatant by dividing it evenly and passing it through two gravity columns kept in a 4 °C cold room, which contained 5 ml of Ni-NTA beads (CAT#H-350-100, GoldBio). Each column had been equilibrated with 20 ml of lysis buffer. After the addition of the supernatant, each column was washed with 20 ml of the lysis buffer, then 20 ml of a wash buffer, which contained 500 mM NaCl, 20 mM Tris at pH 7.5, 60 mM imidazole at pH 7.5, and 1 mM 2-mercaptoethanol. The PLD1 protein was finally eluted from each column using 10 ml of elution buffer, consisting of 150 mM NaCl, 20 mM Tris pH 7.5, 300 mM imidazole, and 1 mM 2-mercaptoethanol, and both 10 ml elutions were pooled together into a single 20 ml elution.

To further purify the PLD1 construct, the Ni-NTA elution was then subjected to size exclusion chromatography. First, the Ni-NTA elution was concentrated in a 30 kDa 15 ml concentrative spin column (REF UFC903024, MilliporeSigma) with a series of 5 min, 4000*g* spins at 4 °C until the total volume was 10 ml. This 10 ml of concentrated supernatant was passed through a 0.45 um filter (Cat No. 13-1001-05, Fisher Scientific) and loaded into a 10 ml sample loop. It was then injected onto a Hiload 26/600 Superdex 200 pg (Cytiva) and the liquid phase was 150 mM NaCl, 20 mM HEPES at pH 7.5, and 10 mM 2-mercaptoethanol. Elution fractions containing the purified PLD1 protein were then pooled together and concentrated by a series of 5 min, 4000*g* spins at 4 °C in a 30 kDa 15 ml concentrative spin column (REF UFC903024, MilliporeSigma). The concentration of the protein was monitored by measuring absorbance at 280 nm with a baseline correction of 340 nm and once the volume had reached 1 ml, the concentrate was then aliquoted and flash-frozen in liquid nitrogen before being stored at −80 °C.

### PLD1 activity assay

To prepare the fluorescent liposome substrate of these activity assays, a 45:45:5:5 molar ratio of POPC (850457, Avanti Polar Lipids), POPE (850757, Avanti Polar Lipids), 14:0/12:0 NBD-PC (810123, Avanti Polar Lipids), and Brain PI(4,5)P_2_ (840046, Avanti Polar Lipids), all prepared in chloroform, were added to the bottom of a glass tube and dried under a stream of gaseous N_2_. The dried lipids were then resuspended in a solution of 100 mM HEPES pH 7.5, 160 mM KCl, 3 mM EGTA, 4 mM CaCl_2_, 6 mM MgCl_2_ (25, 30) such that the total lipid concentration was 1.1 mM. This solution was then vortexed and sonicated for 2 min in a water bath sonicator (Branson 2800) set to “high” to resuspend the dried lipids. The solution was then transferred to a 1.5 ml Eppendorf tube, the Eppendorf tube was buoyed in room temperature water, and tip-sonicated with a micro tip (FB120, FisherBrand) at an intensity of 35% amplitude, 2 s on, 2 s off, for 1.5 min to induce liposome formation (72). After sonication, the solution of fluorescent liposomes was stored in the dark at room temperature until needed.

To prepare the ARL11 proteins for activity assays, aliquots of purified protein were thawed on ice before being centrifuged at 26,670*g* and 4 °C for 10 min to pellet any denatured protein. Then, we performed the following nucleotide loading procedure, which was based on Hammond *et al.* (28), to prepare a solution of 20 μM nucleotide-loaded ARL11, 5 mM EDTA pH 7.5, 10 mM MgCl_2_, and 1 mM GTP. First, the thawed and centrifuged ARL11 is mixed with its size exclusion buffer (150 mM NaCl, 20 mM Tris at pH 7.5, 10 mM 2-mercaptoethanol, and 1 mM MgCl_2_) and EDTA. This mixture was incubated at room temperature for 30 min before the GTP was added, then MgCl_2_ was added, and the exchange reaction was incubated at room temperature for 30 min. The exchange reactions were then left on ice until needed. To prepare GDP-loaded ARL11, GTP was exchanged for GDP in the procedure described above. To make control exchange reactions lacking ARL11, the missing volume which would have been provided by purified ARL11 was substituted with an equal volume of the ARL11 size exclusion buffer (150 mM NaCl, 20 mM Tris at pH 7.5, 10 mM 2-mercaptoethanol, and 1 mM MgCl_2_).

To prepare the PLD1 proteins for activity assays, 10 μl aliquots of PLD1 protein were thawed on ice for 10 min before being centrifuged at 20,627*g* and 4 °C for 10 min to pellet any denatured protein. The thawed and centrifuged PLD1 aliquots were then diluted to 0.6 μm in 150 mM NaCl, 20 mM HEPES at pH 7.5, and 10 mM 2-mercaptoethanol and the final volume was such that each activity reaction could receive 12.5 μl of 0.6 μM PLD1 solution.

To prepare the activity assay reactions themselves, 12.5 μl of 0.6 μM PLD1 solution and 12.5 μl of nucleotide-exchanged 20 μM ARL11 solution were mixed in 1.5 ml eppendorf tubes before 25 μl of 1.1 mM fluorescent liposomes were added. This makes the final concentrations of PLD1 and its mutants, ARL11 and its mutants, and the lipids 0.15 μM, 5 μM, and 0.55 mM respectively. These activity reactions were then allowed to run for 1 h while incubated in a 37 °C water bath. Afterwards, each reaction was quenched by the addition of 400 μl of chloroform:methanol (1:1, v:v) and vortexing. To induce phase separation between aqueous and organic phases, 100 μl of water was added before the solutions were mixed and lightly spun down on a desktop centrifuge. Then, using a 100 μl syringe (Hamilton), 100 μl of the organic phase was extracted from each reaction and transferred to a new 1.5 ml eppendorf tube. The organic solution in these tubes was then dried down under a stream of gaseous N_2_, and resuspended in 100 μl of 0.2% formic acid and 1 mM ammonium formate in HPLC-grade MeOH (buffer B) by vortexing. This resuspension was then spun down at 21,100*g* for 10 min at room temperature. 60 μl of supernatant was then taken from each condition for high performance liquid chromatography (HPLC).

To resolve the substrate and product by HPLC 10 μl was taken from each sample and injected onto a Spectra 3 um C8SR 150 × 3 mmID column (Peeke Scientific). The liquid phase began at 5% buffer A (0.2% formic acid and 1 mM ammonium formate in HPLC-grade water) and 95% buffer B, and a flow rate of 0.2 ml/min. The liquid phase then gradually transitioned to 0% buffer A and 100% buffer B over 12 min, before transitioning back to 5% buffer A and 95% buffer B over 30 s to re-equilibrate the column for the following sample. The fluorescence of the eluent was monitored with an excitation wavelength of 470 nm and an emission wavelength of 530 nm. The concentration of NBD-PA present in the sample was determined by comparing the intensity of the fluorescence of the NBD-PA peak in the eluent to the intensity of NBD-PA (810172, Avanti Polar Lipids) peaks from standards of known concentration (Fig. S2). Before converting the area beneath the NBD-PA product peaks into nmol of NBD-PA per nmol of PLD1 per minute, the average area beneath the two blank samples was subtracted from each NBD-PA product peak’s area.

### NBD-PA HPLC standard curve

In order to be able to convert the fluorescence intensity of the NBD-PA product peaks in our PLD activity assays into nanomoles of NBD-PA, we prepared duplicate 50 μl solutions of 5 μM, 10 μM, 15 μM, 20 μM, and 30 μM NBD-PA in 0.2% formic acid and 1 mM ammonium formate in HPLC-grade MeOH (buffer B), and ran these samples by using the same HPLC resolution method described in the preceding paragraph. The total area beneath each sample’s lone NBD-PA peak was plotted against the nmols of NBD-PA injected from 10 μl of the sample to find the relationship between fluorescence intensity and NBD-PA nmols (Fig. S2), and we found that it was a linear relationship described by the following equation, where y is the area under the fluorescence peak and x is the mass of NBD-PA in nmols: y = 1754.86x-28.88.

### Structure prediction

The browser-based AlphaFold 3 (44) server was used to generate protein structure predictions, both alone and in complex. To generate the predicted structures of PLD1B in complex with various small GTPases, the canonical amino acid sequence listed by Uniprot (73) for each protein was used, and a GTP ligand and Mg^2+^ ion were included in each simulation. The Uniprot ID of each protein used for these structure predictions are as follows: ARF1 (P84077), ARF3 (P61204), ARF4 (P18085), ARF5 (P84085), ARF6 (P62330), ARL1 (P40616), ARL2 (P36404), ARL3 (P36405), ARL4A (P40617), ARL4C (P56559), ARL4D (P49703), ARL5A (Q9Y689), ARL5B (Q96KC2), ARL5C (A6NH57), ARL6 (Q9H0F7), ARL8A (Q96BM9), ARL8B (Q9NVJ2), ARL9 (Q6T311), ARL10 (Q8N8L6), ARL11 (Q969Q4), ARL13A (Q5H913), ARL13B (Q3SXY8), ARL14 (Q8N4G2), ARL15 (Q9NXU5), ARL16 (Q0P5N6), ARL17A (Q8IVW1), ARL17B (Q8IVW1), SAR1A (Q9NR31), SAR1B (Q9Y6B6), TRIM23 (P36406), and ARFRP1 (Q13795).

### Sequence alignment

The Uniprot ID for each protein used in this alignment is listed immediately above.

### Figure generation

Figure 1, Figure 2 and 3*C*, 6, 7, S1–S5, S7, S8, and S11 were generated in RStudio. Figures were modified and edited at Photopea.com.

## Data availability

Data are available to be shared upon request. Please address data requests to Michael V. Airola: michael.airola@stonybrook.edu.

## Supporting information

This article contains supporting information (44, 74, 75, 76, 77).

## Conflict of interest

The authors declare that they have no conflicts of interest with the contents of this article.
