# Supplementary material for: Structural insights into the activation of phospholipase D1 by the small GTPase ARL11
Source: J Biol Chem. 2026 Jul 28;302(9):113375. doi: 10.1016/j.jbc.2026.113375 (PMC13545423; doi:10.1016/j.jbc.2026.113375)
Supplement: Supplementary file 1 [file mmc1.pdf]

SUPPLEMENTAL INFORMATION

Structural Insights into the Activation of Phospholipase D1 by the Small GTPase ARL11

Douglas J. Marr[1], Shujuan Gao[1], Michael A. Frohman[2], Michael V. Airola[1*]

[1] Department of Biochemistry and Cell Biology, Stony Brook University, Stony Brook NY 11794, USA

[2]Department of Pharmacological Sciences, Stony Brook University, Stony Brook, NY, 11794, USA

*Address correspondence to:

Michael V. Airola, michael.airola@stonybrook.edu

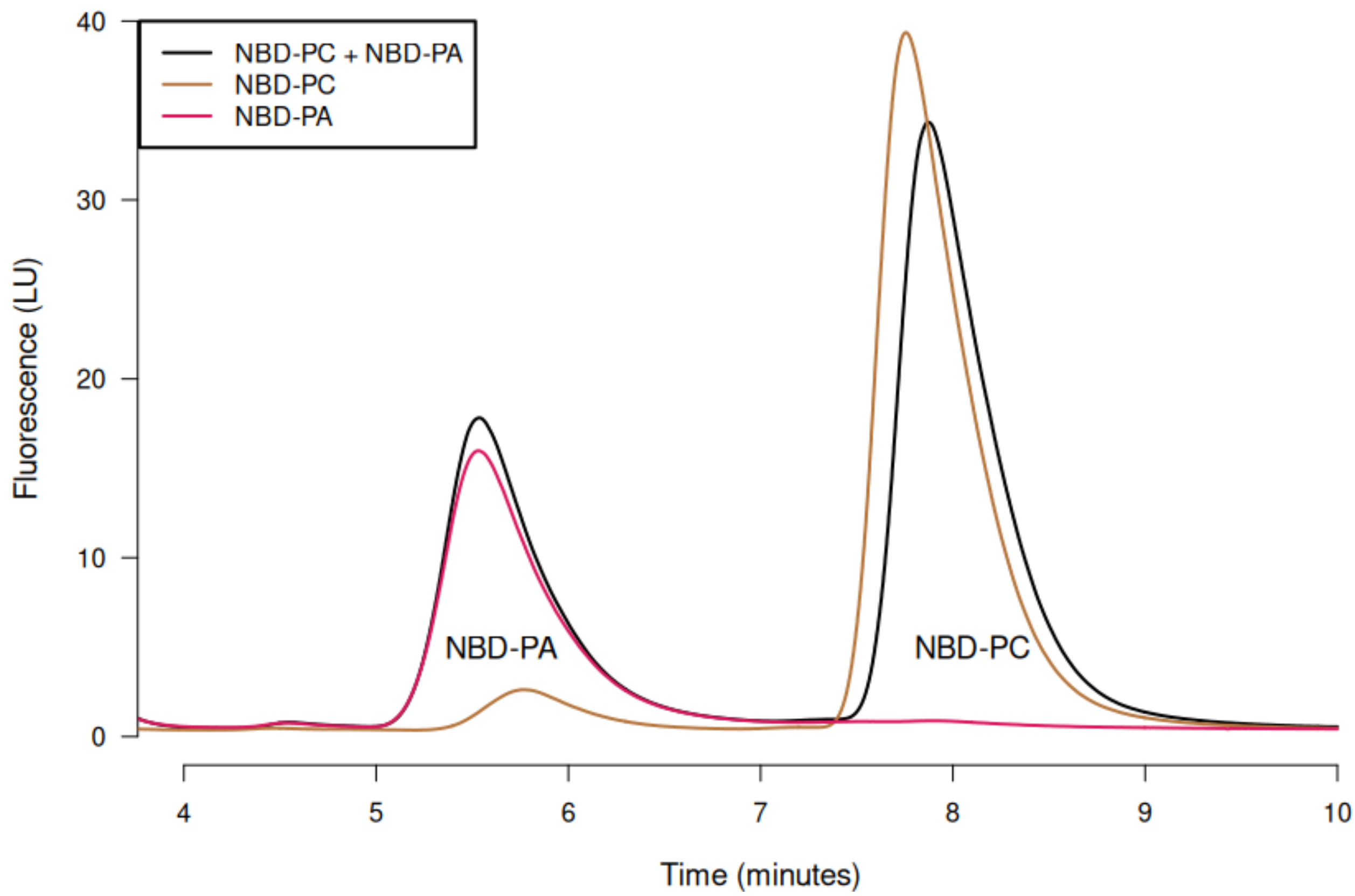

Figure S1. Separation of Fluorescently-Labeled NBD-PC and NBD-PA by HPLC. Overlay of chromatograms in which the fluorescence of the HPLC column eluent is plotted against time in minutes. Fluorescence was measured with an excitation wavelength of 470 nm and an emission wavelength of 530 nm and is reported in Luminescence Units (LUs). Samples contained either 30 μM NBD-PA, 8.89 μM NBD-PC, or both.

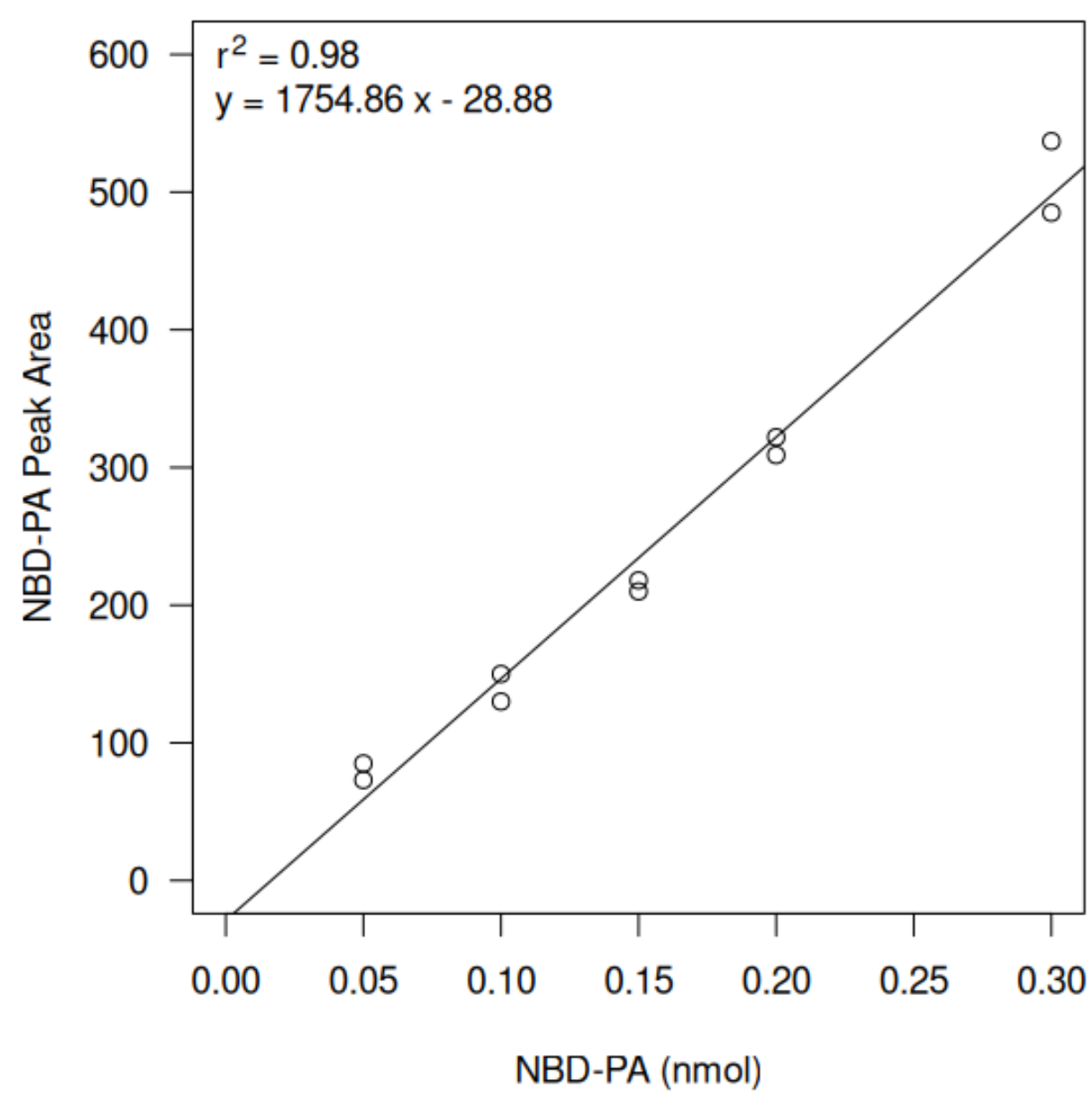

Figure S2. **Standard Curve of NBD-PA Fluorescence at Experimentally Relevant Masses.** The y axis indicates the area beneath fluorescence peaks of NBD-PA detected in the HPLC elution. The relationship between the area under the NBD-PA peak and the mols of NBD-PA in the sample was found to be linear, with the r squared assessment of linearity and equation of the line shown on the plot. This graph depicts a single experiment where each data point represents a single technical replicate.

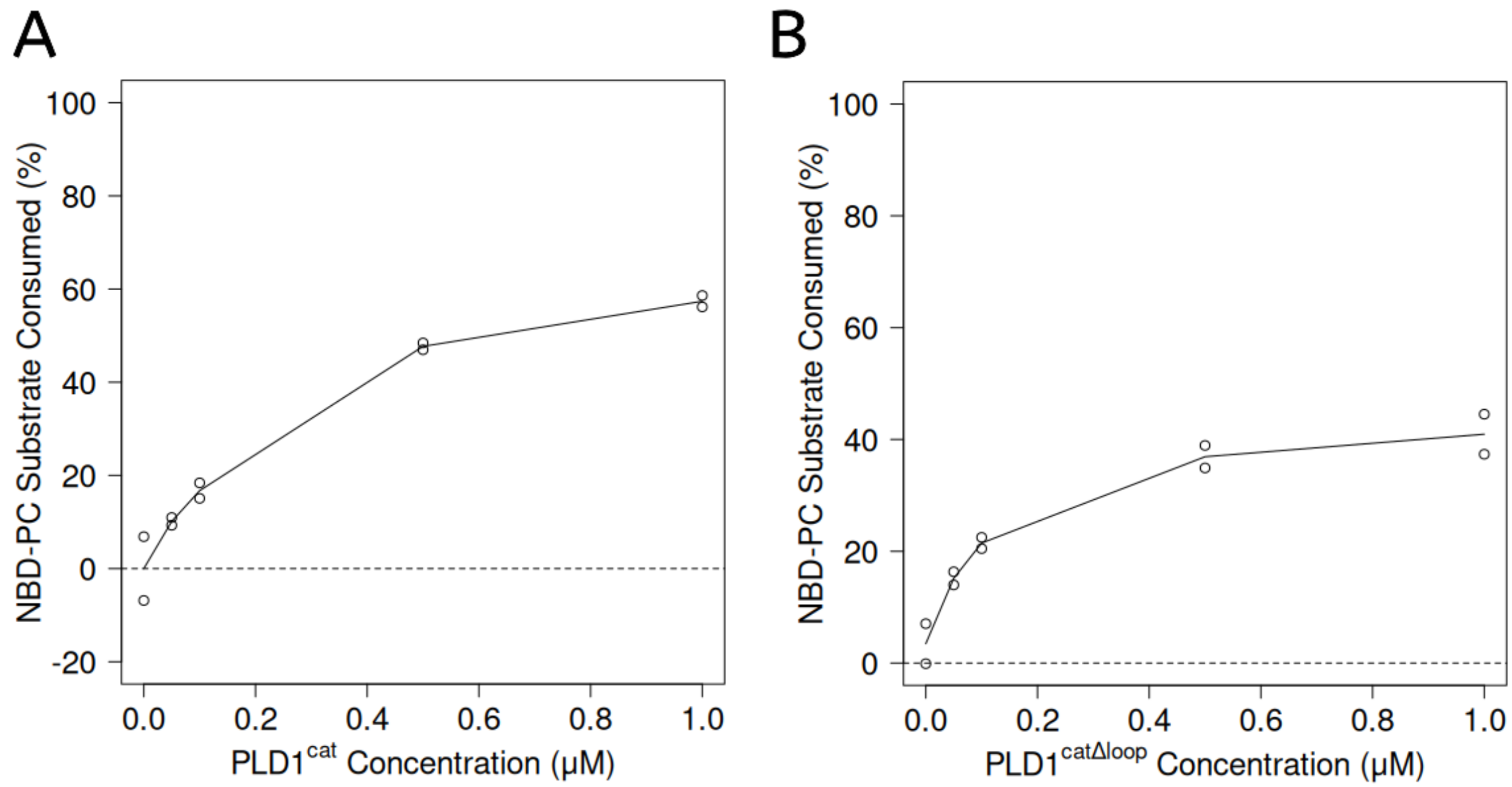

Figure S3. Activity Assay Linear Ranges for $PLD1^{cat}$ and $PLD1^{cat\Delta loop}$ Proteins.

(A) Plot of the percentage of NBD-PC consumed as a function of $PLD1^{cat}$ concentration. NBD-PC consumption scales linearly across the 0-0.1 μM range. Each data point represents a single technical replicate, and the graph depicts data from a single experiment. **(B)** Plot of the percentage of NBD-PC consumed as a function of $PLD1^{cat\Delta loop}$ concentration. NBD-PC consumption scaled linearly across the 0.0-0.1 μM range, but begins to decrease at concentrations above 0.1 μM. Each data point represents a single technical replicate, and the graph depicts data from a single experiment.

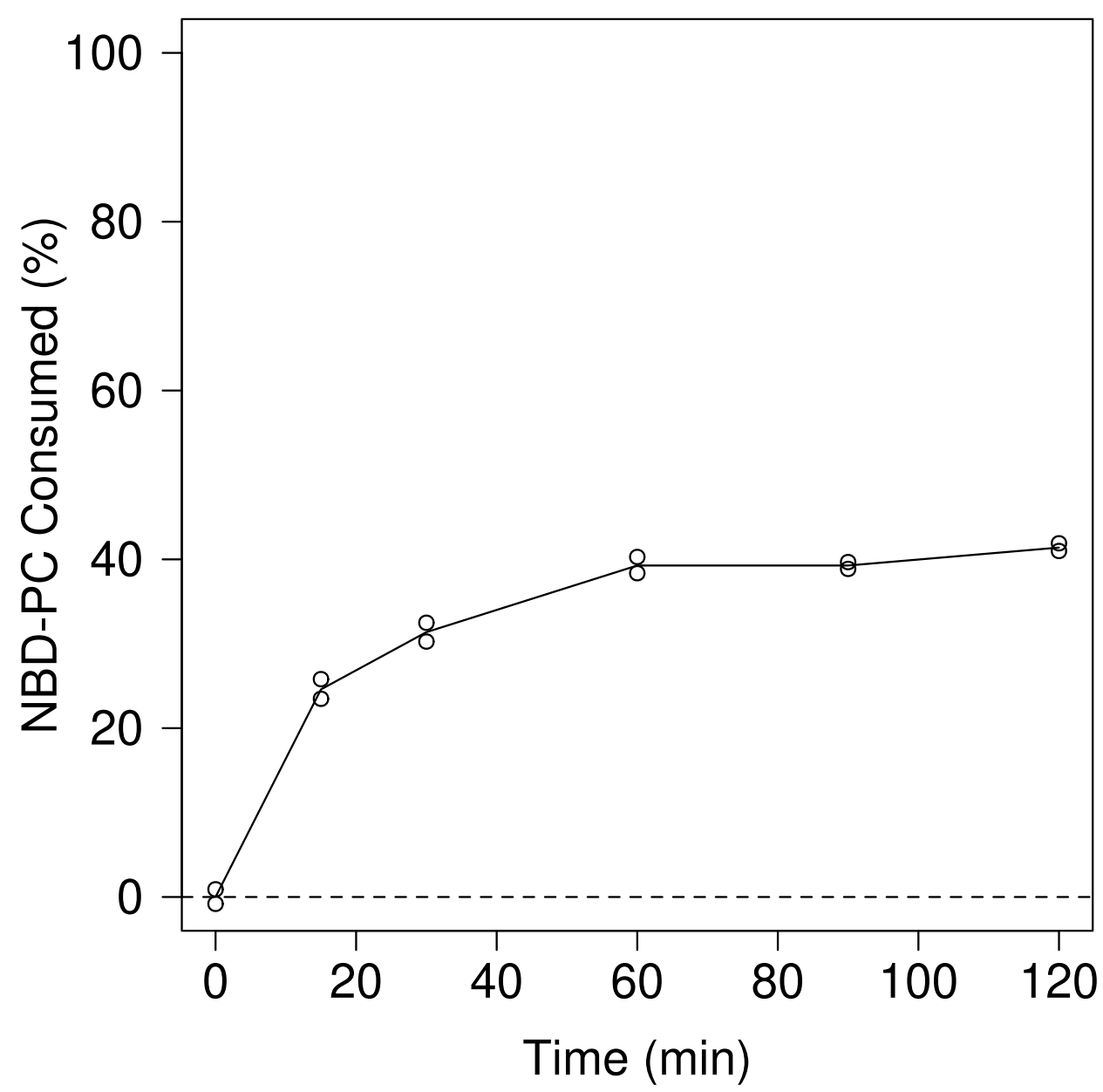

Figure S4. PLD1^{cat} activity over time. Plot of the percentage of NBD-PC consumed over time. Each data point represents a single technical replicate, and each graph depicts data from a single experiment.

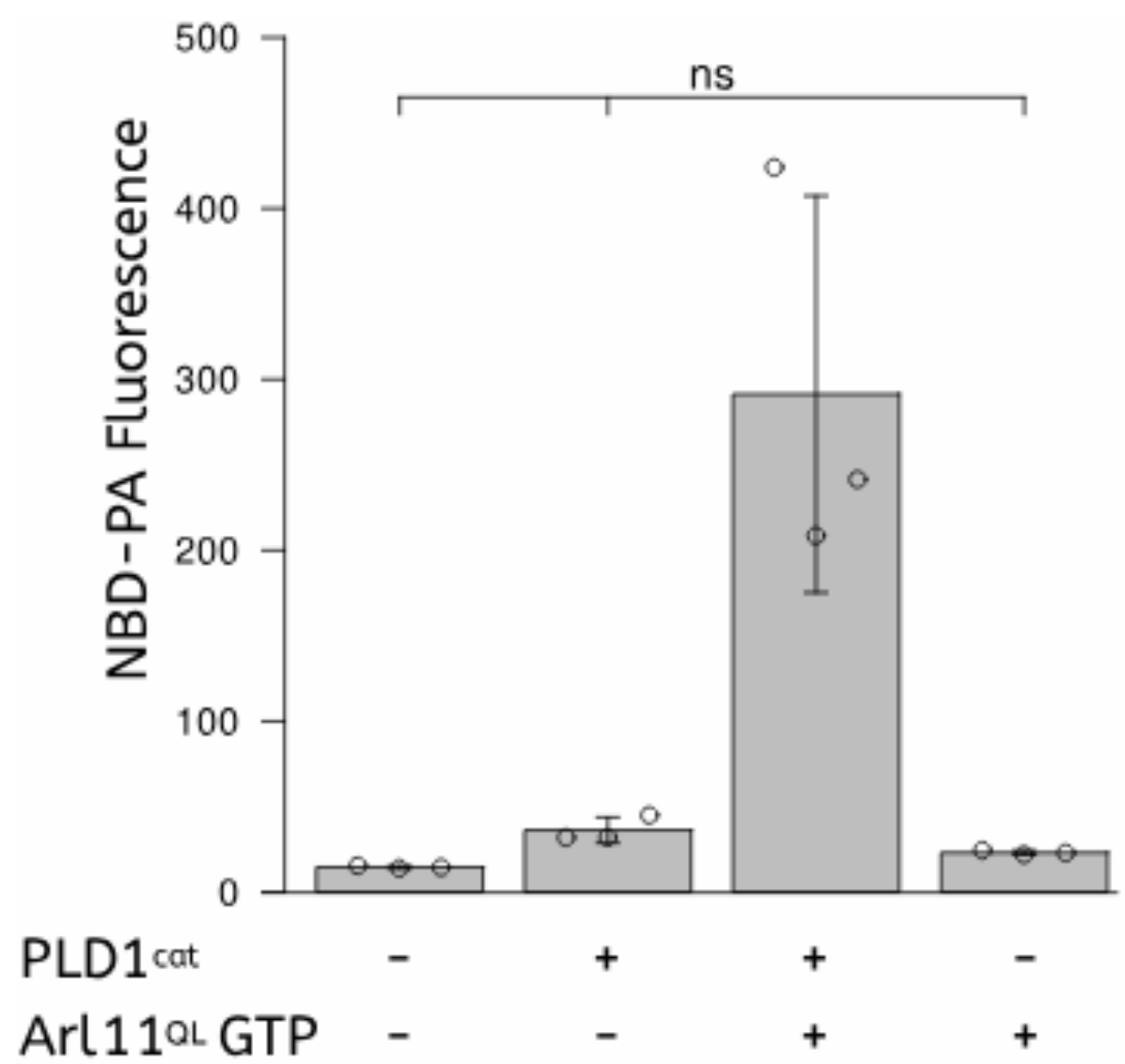

Figure S5. ARL11^{QL} Loaded With GTP is Incapable of Hydrolyzing Phosphatidylcholine in the Absence of PLD1^{cat}. *In vitro* PLD activity assay where the activity of PLD1^{cat} and GTP-loaded ARL11^{QL} are both tested alone and together. The concentration of PLD1^{cat} is 0.15 μM, and the concentration of ARL11^{QL} loaded with GTP is 5 μM. Each datapoint represents the average of two technical replicates, each bar's value is the average of 4 experimental replicates, and the error bars represent the standard deviation of the 4 experimental replicates. Pairwise significance was determined by a Tukey's honest significance test following a one-way ANOVA. The p value at a 95% confidence interval is reported as an abbreviation, where "ns" indicates $p \geq 0.05$.

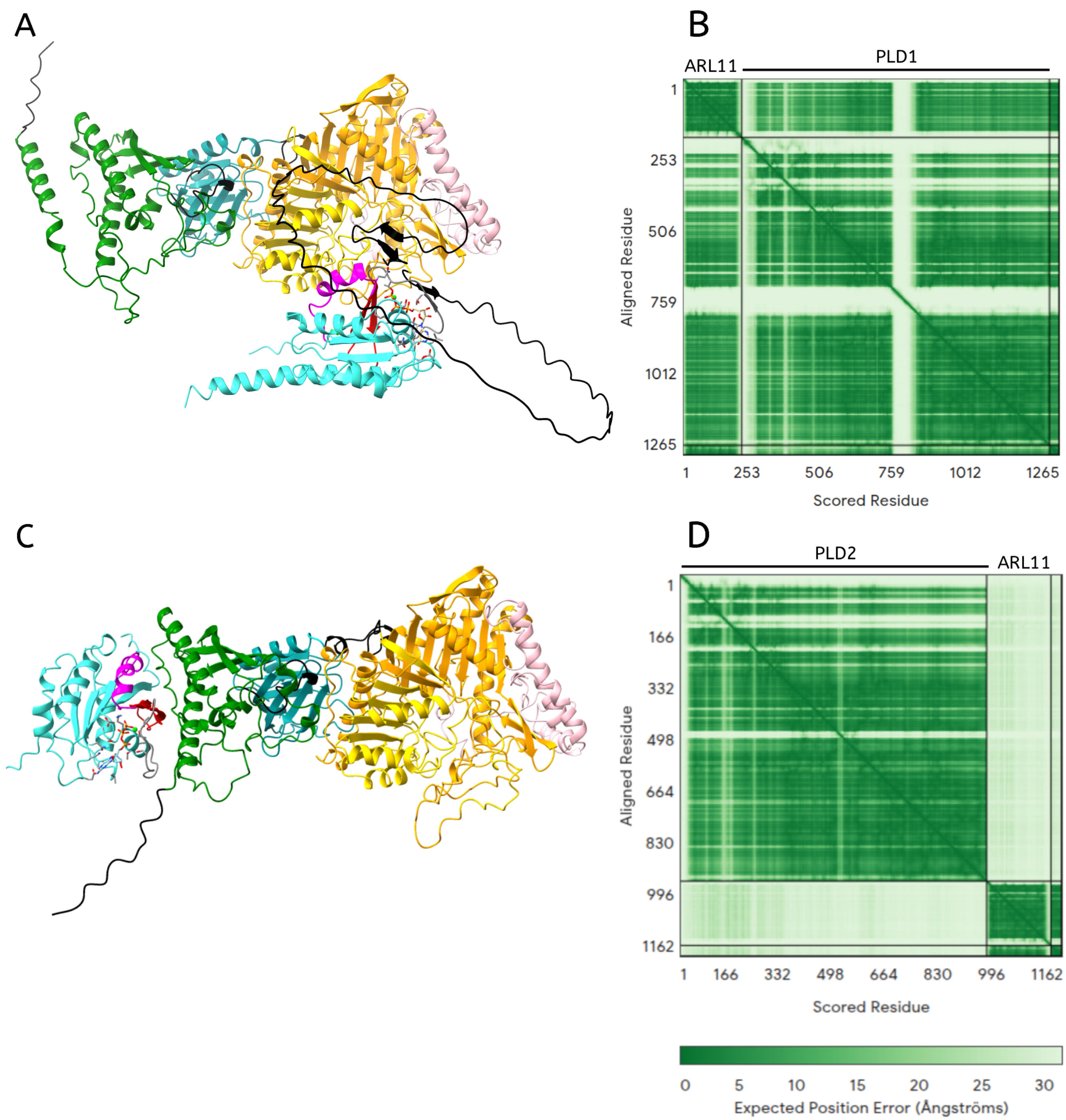

Figure S6. Comparison of PLD1B-ARL11 and PLD2A-ARL11 Structure Predictions.

(A) Structure of the complex between PLD1B and ARL11, including Mg^{2+} (bright green) and GTP (sticks), as predicted by AlphaFold 3[44] and viewed with ChimeraX[73]. The amino acids of each protein have been colored according to the domain they belong to and their depiction in Fig 3A.

(B) The Predicted Aligned Error (PAE) graph generated by AlphaFold 3 for the predicted complex between PLD1B and ARL11. High degrees of confidence are indicated in green and support the relative orientation of ARL11 and PLD1B in the predicted complex. The low confidence regions near residues

200 and 750, conveyed by a lighter green color, correspond to the PLD1B N-terminus and most of its disordered loop, respectively.

(C) Alphafold 3 structure prediction of human phospholipase D2 A (PLD2A) in complex with ARL11, as viewed by ChimeraX. A Mg^{2+} ion (bright green) and GTP (sticks) are also included in the simulation. ARL11 is colored according to its domain architecture as depicted in figure 3A. PLD2A is colored using the same colors as PLD1B in panel A, and these colors have been applied to the domains of PLD2A according to their homology with the domains of PLD1B. The only exception is that PLD2A does not have a lengthy disordered loop between its HKD1 and HKD2 domains. Unlike the prediction for PLD1B, ARL11 is predicted to associate with PLD2A's N-terminal PX domain.

(D) The Predicted Aligned Error (PAE) graph generated by AlphaFold 3 for the predicted complex between PLD2A and ARL11. Lower predicted positional errors are portrayed as a darker green color, while higher predicted positional errors are portrayed as a lighter green color. The entirety of ARL11's amino acid sequence is shown as having high predicted positional aligned error relative to the entirety of PLD2A's amino acid sequence, suggesting that the position of PLD2A and ARL11 relative to each other in this prediction is unlikely to reflect a biological reality.

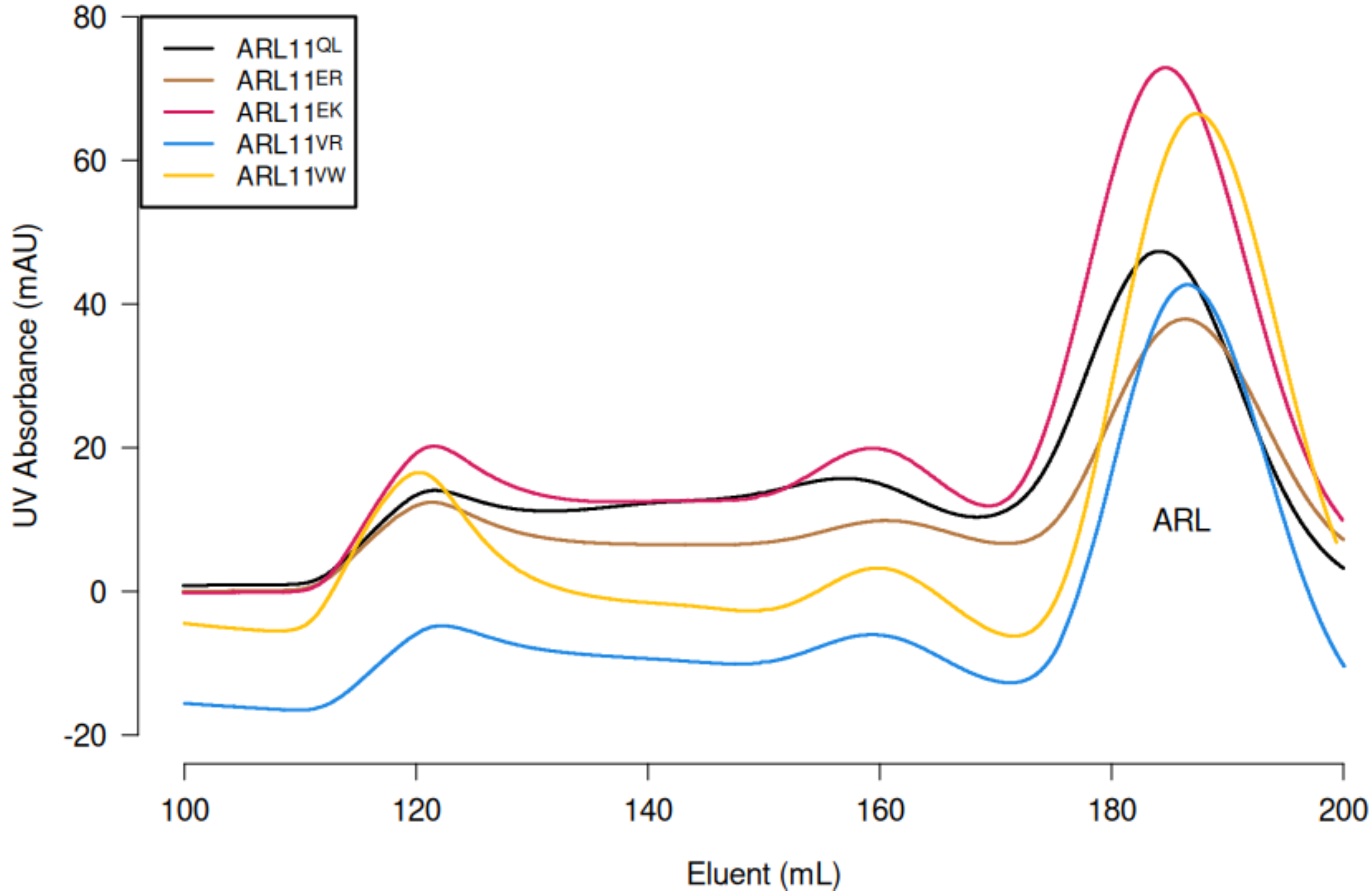

Figure S7. Size Exclusion Chromatography Elution Profiles for ARL11^{QL} and its Point Mutants. This graph shows the UV absorbance in milli absorbance units of each ARL11 mutant plotted against the eluent volume of each run. The rightmost peak, marked with "ARL" was collected, concentrated, and frozen for each protein purification. Each curve represents the data from a single purification, and their UV absorbance values have not been normalized.

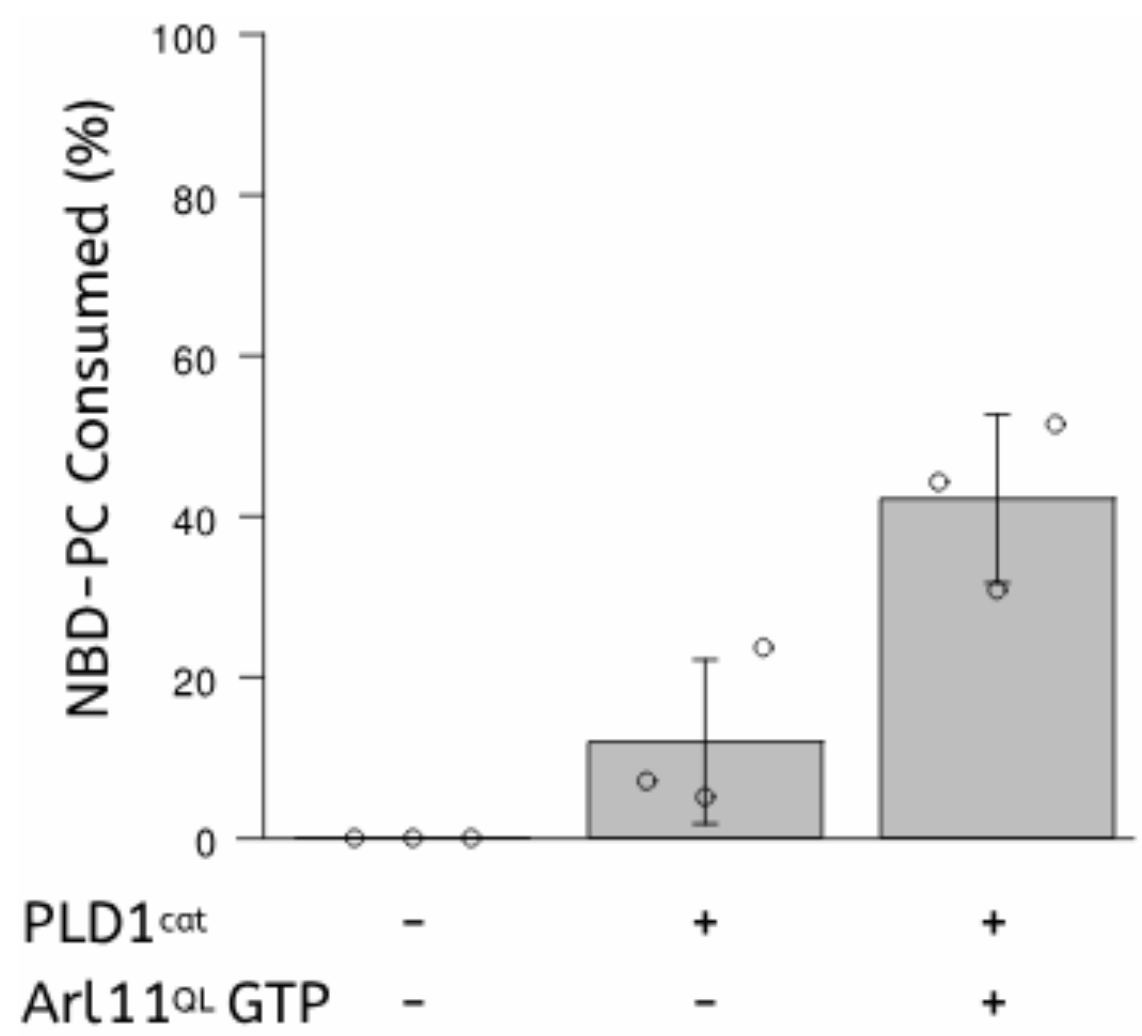

Figure S8. **PLD1^{cat} Stimulated by ARL11^{QL} Sometimes Consumed Nearly all of the Available Substrate.** *In vitro* PLD activity assay where the activity of PLD1^{cat} is tested alone or while exposed to GTP-loaded ARL11^{QL}. The y axis displays PLD1 activity as a percentage of the remaining NBD-PC substrate after incubation with the indicated proteins. This graph was made from reformatting the same data used for Figure 6. Each data point represents the average of two technical replicates, each bar's value is the average of 3 experimental replicates, and the error bars represent the standard deviation of the 3 experimental replicates.

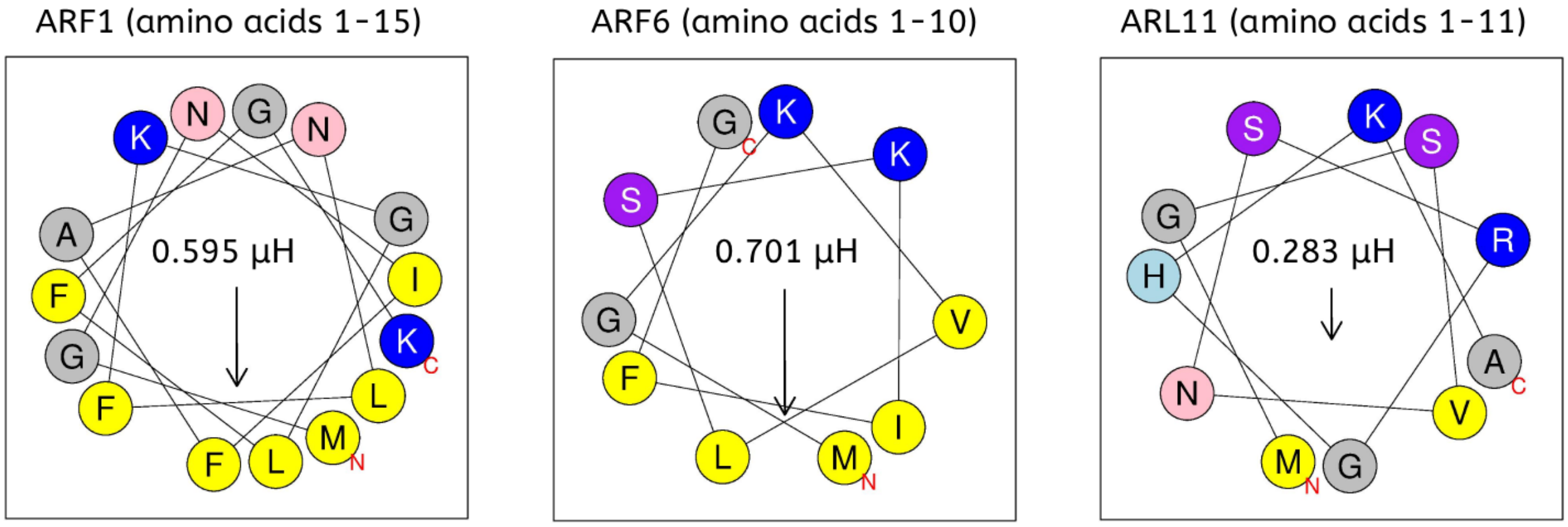

Figure S9. Helical Wheel Diagrams of the N-Termini of ARF1, ARF6, and ARL11. Helical wheel diagrams that depict the primary amino acid sequences of ARF1 (left), ARF6 (middle), and ARL11 (right) at their N-termini. The magnitude and direction of the hydrophobic moment for each sequence is indicated as a vector inside of each wheel. ARL11 is not predicted to form an N-terminal alpha helix by AlphaFold 3. All of these proteins possess the secondary glycine necessary for N-terminal myristoylation. The helical wheel diagrams were generated at the HELIQUEST web server[76]

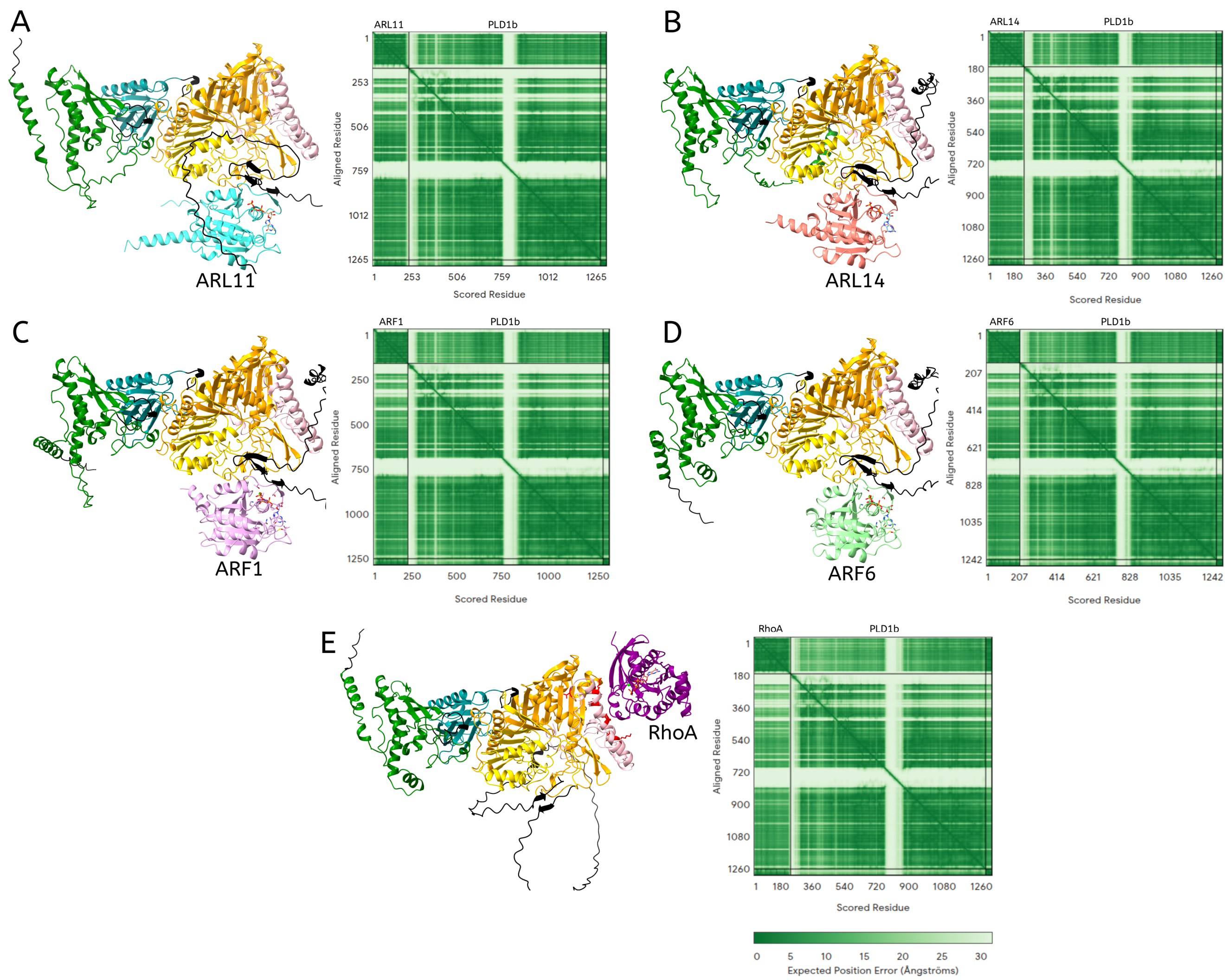

Figure S10. Complexes of PLD1 with ARL11, ARL14, ARF1, ARF6, or RhoA Predicted by AlphaFold. Structure of PLD1 in complex with different known activators, as predicted by AlphaFold 3[44] and visualized in ChimeraX[73]. PLD1 is colored according to its domain architecture (Fig 3A). Each structure prediction was simulated with one molecule of GTP (sticks), and one Mg^{2+} ion (green). The predicted aligned error (PAE) plot for each complex is shown to the right of each structure prediction. **(A)** ARL11 is shown in turquoise. **(B)** ARL14 is shown in salmon. **(C)** ARF1 is shown in light purple. **(D)** ARF6 is shown in light green. **(E)** RhoA is shown in purple. Residues that have been previously identified to disrupt the stimulation of PLD1 by RhoA[77,78] are shown in red. The color gradient conveying confidence at each residue-residue position on the PAE plots is shwon beneath RhoA's PAE plot. High degrees of confidence are indicated in green and support the relative orientation of the proteins in the predicted complex.

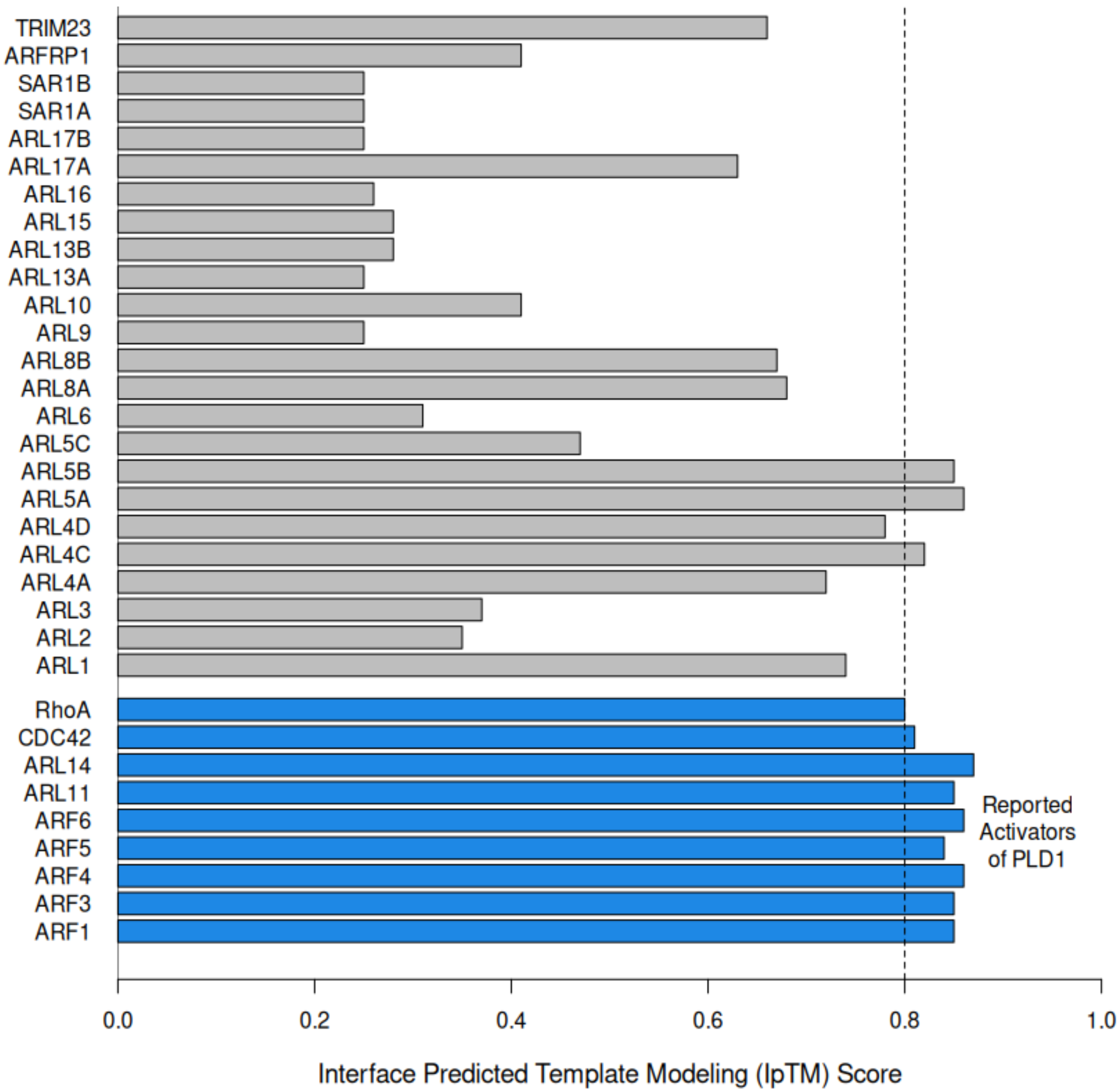

Figure S11. Comparison of Interface Protein Template Modeling Scores Between all ARF GTPases, CDC42 and RhoA, and PLD1B. Graph of the IpTM score for each member of the ARF-family of small GTPases, CDC42, and RhoA when predicted in-complex with PLD1B by AlphaFold 3. Scores greater than 0.8, indicated by a dashed line above, indicate a complex prediction that is likely to reflect the complex's true structure. Proteins reported to activate PLD1 are depicted with blue bars, and separated from the other ARF-family members. RhoA and CDC42 have been reported to activate PLD1, and, therefore, were included in the AlphaFold 3 predictions. ARL4C, ARL5A, and ARL5B exceed the 0.8 standard, and their complexes with PLD1 resemble that of ARL11's (Supplemental data).
